# Gradual reduction in rRNA transcription triggers p53 acetylation and apoptosis via MYBBP1A

**DOI:** 10.1038/srep10854

**Published:** 2015-06-05

**Authors:** Takuya Kumazawa, Kazuho Nishimura, Naohiro Katagiri, Sayaka Hashimoto, Yuki Hayashi, Keiji Kimura

**Affiliations:** 1 Graduate School of Life and Environmental Sciences, University of Tsukuba, 1-1-1 Tennnoudai, Tsukuba 305-8577, Japan; 2 First Department of Internal Medicine, Nara Medical University, 840 Shijo-cho Kashihara, Nara 634-8522, Japan

## Abstract

The nucleolus, whose primary function is ribosome biogenesis, plays an essential role in p53 activation. Ribosome biogenesis is inhibited in response to cellular stress and several nucleolar proteins translocate from the nucleolus to the nucleoplasm, where they activate p53. In this study, we analysed precisely how impaired ribosome biogenesis regulates the activation of p53 by depleting nucleolar factors involved in rRNA transcription or rRNA processing. Nucleolar RNA content decreased when rRNA transcription was inhibited. In parallel with the reduced levels of nucleolar RNA content, the nucleolar protein Myb-binding protein 1 A (MYBBP1A) translocated to the nucleoplasm and increased p53 acetylation. The acetylated p53 enhanced p21 and BAX expression and induced apoptosis. In contrast, when rRNA processing was inhibited, MYBBP1A remained in the nucleolus and nonacetylated p53 accumulated, causing cell cycle arrest at the G1 phase by inducing p21 but not BAX. We propose that the nucleolus functions as a stress sensor to modulate p53 protein levels and its acetylation status, determining cell fate between cell cycle arrest and apoptosis by regulating MYBBP1A translocation.

Mutations in the tumour suppressor p53 gene occur in approximately 50% of all human cancers and play a pivotal role in tumour progression. p53 is maintained at low levels in unstressed cells by its interaction with E3 ubiquitin ligases such as HDM2^1^,^2^,^3^. However, p53 is activated in response to cell stress, producing a number of downstream effects^1^,^4^,^5^,^6^,^7^,^8^. p53 induces cell cycle arrest by activating target genes such as the CDK inhibitor *p21CDKN1A*^9^,^10^ and induces apoptosis by activating distinct classes of target genes, including *BBC3* (*PUMA*)^11^, *BAX*^12^, *PMAIP1* (*NOXA*)^13^, *P53AIP1*^14^ and *TP53I3* (*PIG3*)^15^.

Several studies have reported the mechanisms that select p53 target genes and determine cell fate. First, the strength of p53 binding to cell cycle gene responsive elements (REs) is generally more robust compared with that of apoptosis-related genes^16^. Second, post-translational modifications, such as acetylation of C-terminal lysine residues^17^,^18^, acetylation of the DNA binding domain at Lys-120^19^,^20^ and phosphorylation at Ser-46^14^,^21^,^22^, regulate sequence-specific DNA binding of p53. Third, several p53 binding partners alter the ability of p53 to recognise specific REs and recruit transcriptional coactivators to certain loci^23^,^24^,^25^. Fourth, protein levels of p53 affect the expression of its target genes^26^. Finally, epigenetic histone modifications of p53 target promoters can influence the selection of p53 target genes^27^. These results demonstrate the mechanisms of how p53 produces cellular outcomes under specific stressors. However, the upstream regulators that coordinate the activation of p53 and integrate the stress response into individual outcomes have not been completely elucidated.

Recent studies have revealed that the nucleolus plays an important role in activating p53^28^,^29^. The nucleolus is a distinct subnuclear component that was originally considered the site of ribosome biogenesis, as it functions in ribosomal RNA (rRNA) transcription, rRNA processing and ribosome assembly with many ribosomal proteins. In response to cellular stress, several nucleolar proteins, including p14/p19^Arf^, nucleophosmin, nucleolin, nucleostemin, RPL11, RPL5, RPL23 and RPS7, translocate from the nucleolus to the nucleoplasm, where they activate p53 by inhibiting HDM2^30^,^31^,^32^,^33^,^34^,^35^,^36^,^37^,^38^,^39^,^40^,^41^,^42^. In particular, RPL11 and RPL5, which are components of the 5 S ribonucleoprotein particle (RNP) complex, play an important role activating p53 under conditions in which ribosomal biogenesis is inhibited^43^,^44^,^45^. We recently reported that 5 S RNP activates p53 and induces cellular senescence in response to aberrant ribosome biogenesis under oncogenic and replicative stresses^46^.

In addition, we have shown that nucleolar protein Myb-binding protein 1A (MYBBP1A) acts as a nucleolar stress response molecule by regulating p53 acetylation^47^. Nucleolar localisation of MYBBP1A depends on nucleolar RNA content. Inhibited rRNA transcription following DNA damage or glucose starvation leads to reduced nucleolar RNA content and translocation of MYBBP1A from the nucleolus to the nucleoplasm, where it binds nonacetylated p53, facilitates the interaction between p53 and histone acetyltransferase p300 and induces p53 acetylation and tetramerisation^47^,^48^,^49^,^50^. Then, MYBBP1A dissociates from the acetylated form of p53.

Here we show that enhanced p53 acetylation via MYBBP1A correlated with the induction of apoptosis. MYBBP1A translocated from the nucleolus to the nucleoplasm when rRNA transcription was inhibited, in parallel with a reduction in nucleolar RNA content and elevated p53 acetylation. Acetylated p53 induced apoptosis, accompanied by high p21 and BAX expression levels. In contrast, nucleolar RNA content increased and MYBBP1A remained in the nucleolus when rRNA processing was inhibited. In this case, a nonacetylated form of p53 accumulated via RPL11, which caused cell cycle arrest at the G1 phase by inducing p21. These results suggest that the reduction in nucleolar RNA content strictly regulates MYBBP1A translocation to the nucleoplasm, thereby determining cell fate between cell cycle arrest and apoptosis.

## Results

### Reduced levels of rRNA transcription correlate with the induction of apoptosis

Impaired ribosome biogenesis in response to cellular stress reportedly leads to the activation of p53^28^,^51^. Activated p53 determines cell fate (e.g. cell cycle arrest or apoptosis) by inducing distinct classes of target genes^1^. However, the relationship between impaired ribosome biogenesis and the p53-mediated cell fate decision remains to be elucidated.

**Figure 1:**
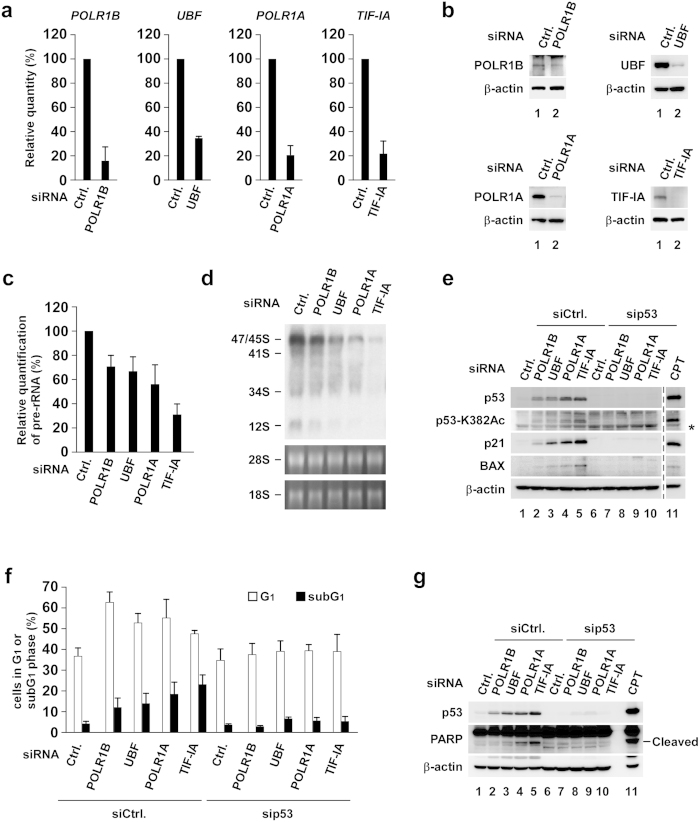
Gradual reduction in rRNA transcription correlates with the activation of p53 and the induction of apoptosis. (**a–d**) MCF-7 cells were transfected with siCtrl, siPOLR1B, siUBF, siPOLR1A or siTIF-IA. (**a**) Expression of rRNA transcription factors was assessed by reverse transcription-quantitative polymerase chain reaction (RT-qPCR) at 48 h after transfection (n = 3). Error bars indicate mean ± standard deviation (SD). (**b**) Protein levels of rRNA transcription factors were assessed by immunoblotting at 48 h after transfection. (**c**) pre-rRNA transcription was assessed by RT-qPCR at 60 h after transfection (n = 3). Error bars indicate mean ± SD. (**d**) Northern blotting was performed at 60 h after transfection using a probe specific for the rRNA internal transcribed spacer 1 region. An ethidium bromide-stained gel is shown at the bottom as a loading control. (**e–g**) MCF-7 cells were transfected with combinations of siRNAs as indicated. (**e**) Cell lysates were prepared at 60 h after transfection and immunoblotted using the indicated antibodies. Lysates from 2 μg/ml camptothecin (CPT)-treated cells were used as a positive control. Asterisk indicates nonspecific bands. (**f**) DNA content was determined by flow cytometry at 72 h after transfection (n = 3). Error bars indicate mean ± SD. (**g**) Cell lysates were prepared at 72 h after transfection and immunoblotted using the indicated antibodies. Lysates from 2 μg/ml CPT-treated cells were used as a positive control.

The proteins implicated in ribosome biogenesis were depleted to investigate the relationship between impaired ribosome biogenesis and p53 activation status. First, we depleted several rRNA transcription factors, such as polymerase RNA 1 polypeptide B (POLR1B), upstream binding factor (UBF), polymerase RNA 1 polypeptide A (POLR1A) and transcription initiation factor IA (TIF-IA) in MCF-7 cells (Fig. 1a,b). Downregulating these factors inhibited rRNA transcription, as shown by the reverse transcription-quantitative polymerase chain reaction (Fig. 1c) and Northern blotting analyses using an internal transcribed sequence 1 probe (Fig. 1d). Similar results were obtained in U2OS cells (Fig. S2a and S2b). The reduced rate of rRNA transcription differed among the factors that were depleted (Figs. 1c,d, S2a and S2b). The total amount and K382-acetylation of p53 increased and were correlated with a reduced rRNA transcription rate (Figs. 1c–e and S2a–c). p21 and BAX are also increased in parallel with the activation of p53 and inhibition of rRNA transcription (Figs. 1c–e and S2a–c). Elevated p21 and BAX levels were abrogated by depleting p53 (Figs. 1e and S2c).

Next, we tested the effect of depleting the rRNA transcription factors on cell cycle arrest at the G1 phase or apoptosis because depleting these factors upregulated p21 and BAX protein levels. We showed that depleting these factors using fluorescence-activated cell sorting (FACS) analysis increased the ratios of cells with subG1 DNA content, which was reversed by depleting p53 (Figs. 1f, S1 and S2d). We also found the enhanced poly (ADP-ribose) polymerase (PARP) cleavage, an indicator of apoptosis (Figs. 1g and S2e). Notably, the subG1 apoptotic cell ratios and PARP cleavage were correlated with the reduced rate of rRNA transcription. The cells with G1 DNA content were increased in MCF-7 cells (Fig. 1f) but not in U2OS cells (Fig. S2d). Taken together, these results suggest that the total amount and acetylation of p53 increased, and that activated p53 caused apoptosis in response to a decrease in rRNA transcription.

### Gradual reduction in nucleolar RNA content leads to translocation of MYBBP1A, p53 acetylation and apoptosis in a dose-dependent manner

We reported previously that inhibiting rRNA transcription decreases nucleolar RNA content and causes translocation of the nucleolar protein MYBBP1A to the nucleoplasm, where it enhances K382-acetylation of p53 by stabilising the association between p53 and p300^47^,^48^.

**Figure 2:**
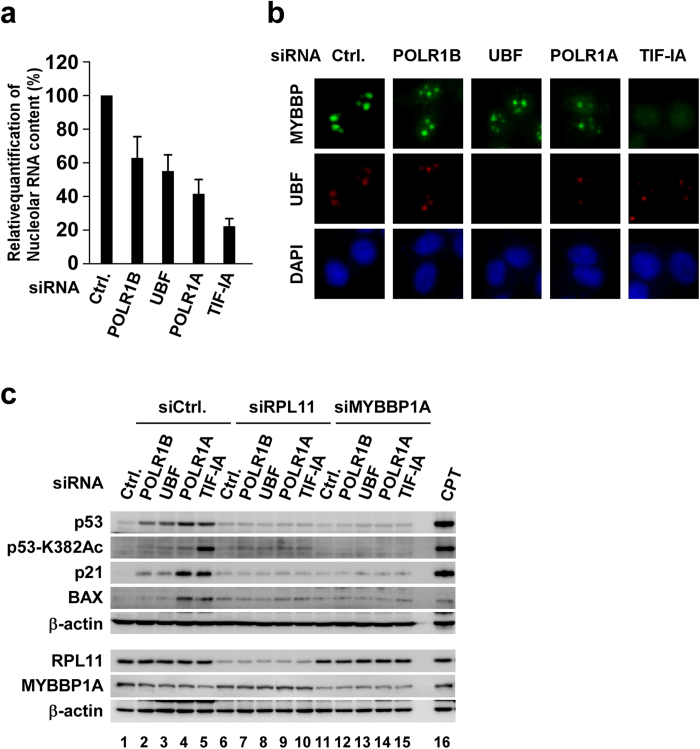
p53 acetylation was enhanced by the nucleolar protein Myb-binding protein 1A (MYBBP1A) in parallel with a reduction in nucleolar RNA content. (**a**) MCF-7 cells were transfected with siCtrl, siPOLR1B, siUBF, siPOLR1A or siTIF-IA. Nucleolar RNA content was spectrophotometrically quantified at 60 h after transfection (n = 3). Nucleolar RNA content of control cells was normalised to 100%. Error bars indicate mean ± standard deviation. (**b**) MCF-7 cells were transfected with the indicated siRNAs. Immunofluorescence staining was performed using the indicated antibodies at 60 h after transfection. Upstream binding factor (UBF) was used as a nucleolar marker. (**c**) MCF-7 cells were transfected with combinations of siRNAs as indicated for 60 h. Cell lysates were prepared from these cells and were immunoblotted using the indicated antibodies. Lysates from 2 μg/ml camptothecin-treated cells were used as the positive control.

In this study, we evaluated whether MYBBP1A enhances p53 acetylation, which was negatively correlated with rRNA transcription levels. We first confirmed that nucleolar RNA content decreased in parallel with the reduction in rRNA transcription when several rRNA transcription factors were depleted in MCF-7 cells (Fig. 2a). Next, we examined subcellular localisation of MYBBP1A. MYBBP1A was localised predominantly in the nucleolus of control cells, whereas it translocated from the nucleolus to the nucleoplasm in response to the depletion of rRNA transcription factors (Fig. 2b). Notably, more MYBBP1A translocated from the nucleolus with lower nucleolar RNA content (Fig. 2a,b).

Then, we tested whether MYBBP1A is required for the gradual activation of p53 when several rRNA transcription factors were depleted, because translocated MYBBP1A activates p53^47^,^48^. As expected, the activation of p53 and the accumulation of p21 and BAX were counteracted by depleting MYBBP1A (Fig. 2c). Depleting RPL11, a component of the 5 S RNP complex, also reversed these phenomena (Fig. 2c). This observation is consistent with reports that 5 S RNP components induce the accumulation of p53 by inhibiting HDM2 and that they are required to translocate MYBBP1A to the nucleoplasm^47^,^48^. Similar results were observed in U2OS cells (Fig. S3). Thus, it is likely that p53 is activated via MYBBP1A and RPL11 in response to impaired rRNA transcription.

**Figure 3:**
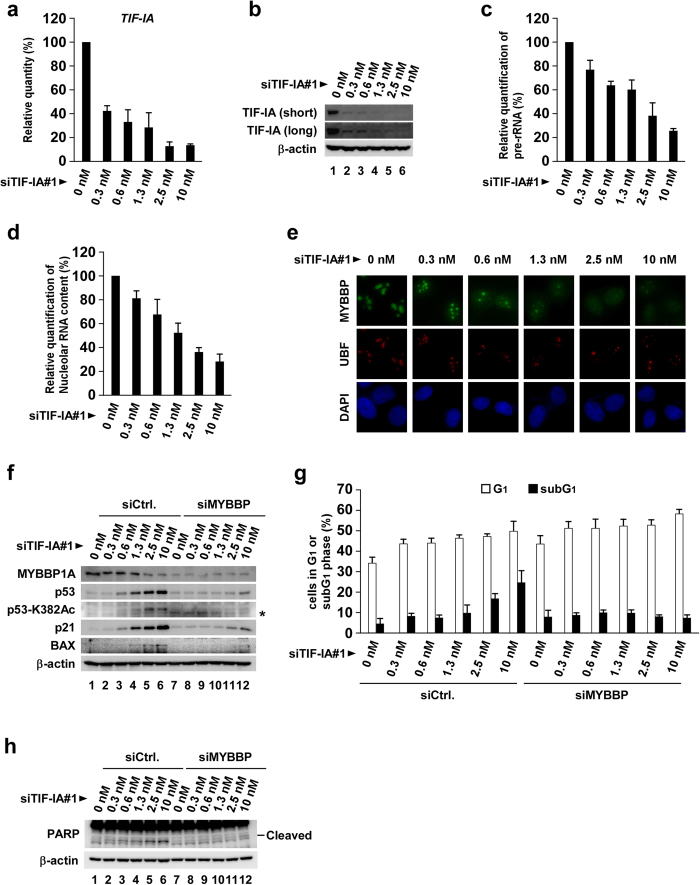
siRNA for transcriptional intermediary factor 1A (TIF-IA) treatment causes Myb-binding protein 1A (MYBBP1A)-dependent p53 acetylation and apoptosis in a dose-dependent manner. (**a–e**) MCF-7 cells were transfected with the indicated concentrations of siTIF-IA. (**a**) TIF-IA mRNA levels were assessed by reverse transcription-quantitative polymerase chain reaction (RT-qPCR) at 60 h after transfection (n = 3). Error bars indicate mean ± standard deviation (SD). (**b**) Protein levels of TIF-IA were assessed by immunoblotting at 36 h after transfection. The middle panel is long exposure of the top panel. (**c**) pre-rRNA transcription was assessed by RT-qPCR at 60 h after transfection (n = 3). Error bars indicate mean ± SD. (**d**) Nucleolar RNA was isolated from purified nucleoli and quantified by spectrophotometry at 60 h after transfection (n = 3). Nucleolar RNA content of control cells was normalised to 100%. Error bars indicate mean ± SD. (**e**) Immunofluorescence staining was performed using the indicated antibodies at 60 h after transfection. (**f–h**) MCF-7 cells were transfected with combinations of siRNAs as indicated. (**f**) Cell lysates were prepared at 60 h after transfection and were immunoblotted using the indicated antibodies. Asterisk indicates nonspecific bands. (**g**) DNA content was determined by flow cytometry at 72 h after transfection (n = 3). Error bars indicate mean ± SD. (**h**) Cell lysates were prepared at 72 h after transfection and were immunoblotted using the indicated antibodies.

We showed that p53 acetylation and the induction of apoptosis correlated with a decrease in nucleolar RNA content when several rRNA transcription factors were depleted (Figs. 1,2, S2 and S3). However, we cannot exclude the possibility that other signals or pathways are involved in the activation of p53 and the induction of apoptosis, besides the reduction in nucleolar RNA content. Therefore, we treated MCF-7 cells with various concentrations of siTIF-IA and tested the relationship between reduced nucleolar RNA content and the activation of p53. TIF-IA levels were negatively correlated with siTIF-IA concentration (Fig. 3a,b). Pre-rRNA levels and nucleolar RNA content also decreased in an siTIF-IA concentration-dependent manner (Fig. 3c,d). MYBBP1A translocated from the nucleolus to the nucleoplasm in proportion to the reduction in nucleolar RNA content (Fig. 3e). The total amounts and acetylation status of p53, p21 and BAX (Fig. 3f), cells in the subG1 phase (Figs. 3g and S4) and PARP cleavage (Fig. 3h) increased in line with the reduced nucleolar RNA content. These phenomena (i.e. the enhancement of total amounts and acetylation of p53, p21, BAX, cells in the subG1 phase and PARP cleavage) were reversed in the presence of siMYBBP1A (Figs. 3f–h and S4). Similar results were observed when MCF-7 cells were treated with another siRNA for TIF-IA (Fig. S5) and U2OS cells were treated with siTIF-IA (Fig. S6). The cells with G1 DNA content were increased by siTIF-IA treatment in MCF-7 cells (Figs. 3g and S5e) but not in U2OS cells (Fig. S6e). Taken together, our results show that MYBBP1A, which translocated to the nucleoplasm in parallel with reduced nucleolar RNA content, activated p53 by enhancing acetylation and induced apoptosis when rRNA transcription was inhibited.

### Depleting the rRNA processing factors results in the accumulation of p53 and G1 cell cycle arrest, which are RPL11 dependent and MYBPB1A independent

**Figure 4:**
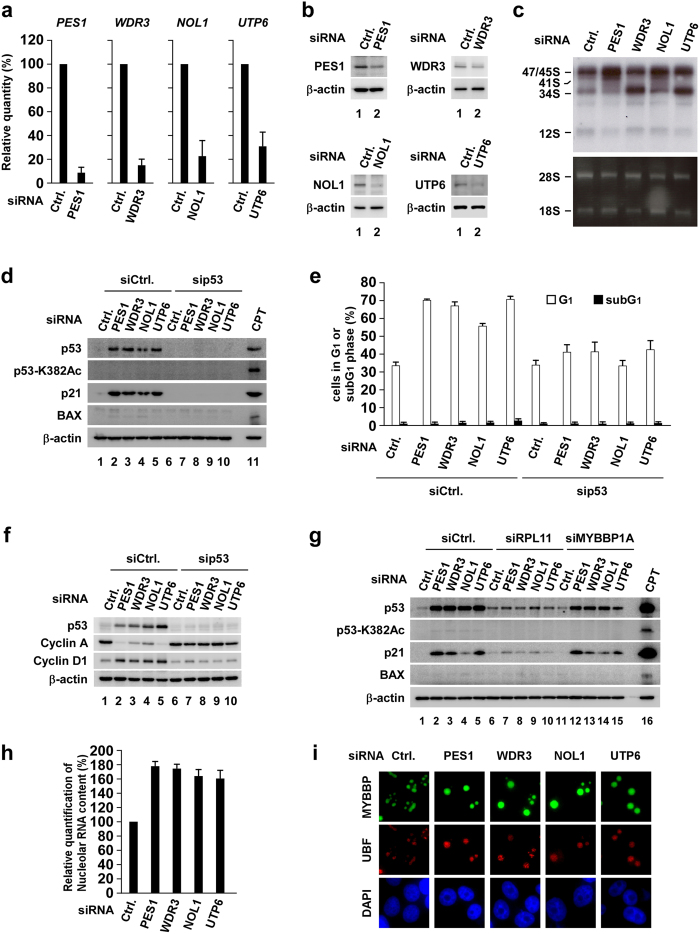
Depleting rRNA processing factors causes RPL11-dependent and Myb-binding protein 1A (MYBBP1A)-independent p53 activation. (**a–c**) MCF-7 cells were transfected with siCtrl, siPES1, siWDR3, siNOL1 or siUTP6. (**a**) Expression of rRNA processing factors was assessed by reverse transcription-quantitative polymerase chain reaction (RT-qPCR) at 48 h after transfection (n = 3). Error bars indicate mean ± SD. (**b**) Protein levels of rRNA processing factors were assessed by immunoblotting at 48 h after transfection. (**c**) Northern blotting was performed at 60 h after transfection using a probe specific for the rRNA region. An ethidium bromide-stained gel is shown at the bottom as the loading control. (**d–g**) MCF-7 cells were transfected with combinations of siRNAs as indicated. (**d**) Cell lysates were prepared at 60 h after transfection and immunoblotted using the indicated antibodies. Lysates from 2 μg/ml camptothecin (CPT)-treated cells were used as the positive control. (**e**) DNA content was determined by flow cytometry at 72 h after transfection (n = 3). Error bars indicate mean ± standard deviation (SD). (**f**) Cell lysates were prepared at 72 h after transfection and immunoblotted using the indicated antibodies. (**g**) Cell lysates were prepared at 60 h after transfection and immunoblotted using the indicated antibodies. Lysates from 2 μg/ml CPT-treated cells were used as the positive control. (**h**) Nucleolar RNA content was spectrophotometrically quantified at 60 h after transfection (n = 3). Nucleolar RNA content of control cells was normalised to 100%. Error bars indicate mean ± SD. (i) Immunofluorescence staining was performed using the indicated antibodies at 60 h after transfection.

Defects in rRNA processing by the depletion of rRNA processing factors, such as PES1, WDR3 and Las1L, reportedly results in the accumulation of p53 and cell cycle arrest at the G1 phase^52^,^53^,^54^. In addition, we have recently found that persistent depletion of several rRNA processing factors induces p53-dependent cellular senescence^46^. To assess the mechanistic difference between impaired rRNA transcription- and impaired rRNA processing-induced p53 activation, we depleted several rRNA processing factors in MCF-7 (Figs. 4 and S7) and in U2OS cells (Fig. S8). We first confirmed that siRNA treatment efficiently reduced these factors (Fig. 4a,b) and induced defects in rRNA processing (Fig. 4c). Depleting rRNA processing factors caused p53 accumulation, p53-dependent p21 induction (Figs. 4d and S8a) and p53-dependent cell cycle arrest at the G1 phase, as shown by FACS analysis (Figs. 4e, S7 and S8b). Consistent with FACS analysis, immunoblotting showed that Cyclin D1, a marker of the G1 phase, increased and Cyclin A, which accumulates in the S, the G2 and the early M phases, decreased by depleting these factors (Figs. 4f and S8c).

Depleting the processing factors scarcely induced K382-acetylation of p53, expression of BAX and apoptosis (Figs. 4d,e, S8a and S8b), which differed from depleting the rRNA transcription factors (Figs. 1, S2–S6). Therefore, we depleted either RPL11 or MYBBP1A with the rRNA processing factors to identify the protein that activates p53 when rRNA processing was inhibited. We found that depleting RPL11 (but not MYBBP1A) compromised the accumulation of p53 and the induction of p21 (Figs. 4g and S8d). This result shows that RPL11 is involved in activating p53 caused by impaired rRNA processing, whereas MYBBP1A is not involved. Consistent with this result, when the rRNA processing factors were depleted, nucleolar RNA content did not drop (Figs. 4h and S8e)^46^ and MYBBP1A was not translocated to the nucleoplasm (Figs. 4i and S8f).

**Figure 5:**
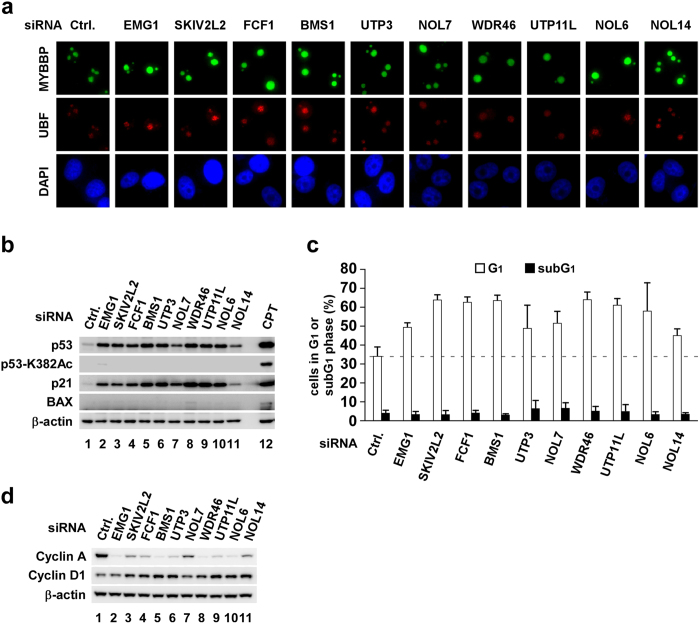
Depletion of various rRNA processing factors activates p53 without translocating Myb-binding protein 1A (MYBBP1A). (**a–d**) MCF-7 cells were transfected with siRNAs against rRNA processing factors. (**a**) Immunofluorescence staining was performed using the indicated antibodies at 60 h after transfection. (**b**) Cell lysates were prepared at 60 h after transfection and immunoblotted using the indicated antibodies. Lysates from 2 μg/ml camptothecin-treated cells were used as the positive control. (**c**) DNA content was determined by flow cytometry at 72 h after transfection (n = 3). Error bars indicate mean ± standard deviation. (**d**) Cell lysates were prepared at 72 h after transfection and immunoblotted using the indicated antibodies.

Next, we depleted 10 other rRNA processing factors in MCF-7 cells. Similar cellular responses, such as retention of MYBBP1A in the nucleolus (Fig. 5a), p53 accumulation (Fig. 5b) and G1 cell cycle arrest (Figs. 5c,d and S9), were observed in these cells, indicating a relationship between MYBBP1A-independent p53 activation and G1 cell cycle arrest. Taken together, these results show that defects in rRNA processing result in the accumulation of p53 and p21 and cell cycle arrest at the G1 phase, which depends on RPL11.

**Figure 6:**
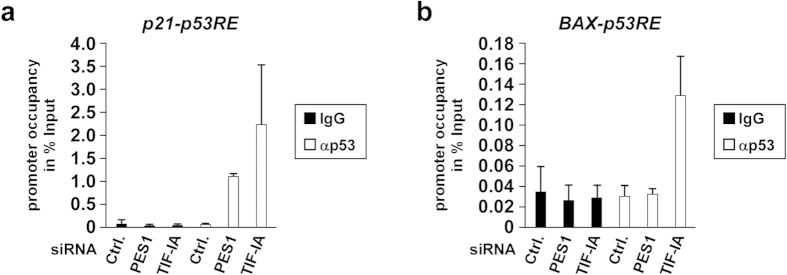
Inhibiting rRNA transcription enhances p53 DNA-binding activity. MCF-7 cells were transfected with siCtrl, siPES1 or siTIF-IA. The chromatin immunoprecipitation assay was performed using normal mouse IgG and anti-p53 antibodies. The p53-binding regions of the *p21* promoter (**a**) or the *BAX* promoter (**b**) were amplified and analysed by reverse transcription-quantitative polymerase chain reaction (n = 3). Error bars indicate mean ± standard deviation.

Finally, we performed a chromatin immunoprecipitation (ChIP) assay to determine why inhibiting rRNA transcription and rRNA processing caused apoptosis and cell cycle arrest. We noticed that p53 bound to the p21 promoter when either TIF-IA, an rRNA transcription factor, or PES1, an rRNA processing factor, were depleted (Fig. 6a). In contrast, p53 bound to the BAX promoter when TIF-IA, but not PES1, was depleted (Fig. 6b). These results indicate that MYBBP1A-mediated K382 acetylation of p53 enhances p53 promoter-binding activity and causes apoptosis when rRNA transcription is inhibited.

## Discussion

**Figure 7:**
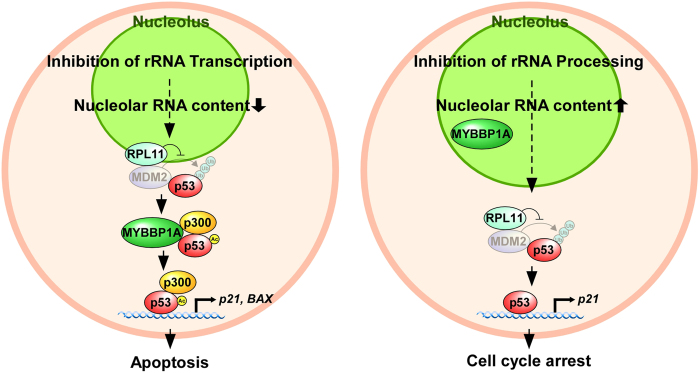
Cell fate decision model regulated by the nucleolus. Inhibiting rRNA transcription enhanced p53 acetylation and apoptosis, whereas inhibiting rRNA processing stabilised p53 and caused G1 cell cycle arrest. See the Discussion for details.

The present data demonstrate that the nucleolus strictly regulates the activation of p53 and subsequent cell fate. Inhibiting rRNA transcription enhanced p53 acetylation and led to apoptosis, whereas inhibiting rRNA processing led to the accumulation of p53 and caused cell cycle arrest at the G1 phase. The nucleolar protein MYBBP1A is indispensable for the former process but not for the latter process (Fig. 7).

Inhibition of rRNA transcription causes p53 activation and apoptosis^42^,^47^. When rRNA transcription was inhibited by depleting rRNA transcription factors, nucleolar RNA content was decreased and the nucleolus disrupted (Figs. 2,3, S3, S5 and S6). Nucleolar RNA content was decreased in a stepwise manner either by depleting several rRNA transcription factors (Figs. 2 and S3) or by depleting an identical rRNA transcription factor (TIF-IA) with various siRNA concentrations (Figs. 3, S5 and S6). In both cases, the reduced nucleolar RNA content was correlated with the MYBBP1A translocation to the nucleoplasm (Figs. 2,3, S3, S5 and S6). Furthermore, the ratio of translocation of MYBBP1A parallelled the upregulation of p53 K382-acetylation (Figs. 2,3, S3, S5 and S6). These results strongly support our previous study^47^,^48^, in which we proposed that MYBBP1A is anchored in the nucleolus by binding to nucleolar RNA and that translocated MYBBP1A enhances p53 acetylation by stabilising the binding between p300 and p53.

The data in Figs. 2c,3f, S5d and S6d show that depletion of rRNA transcription factors reduces the protein levels of MYBBP1A, which apparently opposes the increased p53 acetylation (because MYBBP1A enhances p53 acetylation). However, our previous publication may account for the discrepancy between the decreased MYBBP1A and the enhanced p53 acetylation as follows: in response to nucleolar disruption, MYBBP1A translocated to the nucleoplasm, where it bound to nonacetylated p53, enhanced its acetylation and then dissociated from acetylated p53^50^. Thus, persistent interaction between MYBBP1A and p53 would not be required for p53 acetylation: MYBBP1A may be degraded after it transiently associates with p53 and induces p53 acetylation. This idea is supported by the result that MG132 alleviated the reduction in MYBBP1A protein levels by siTIF-IA treatment (Fig. S10). Analysing the significance of MYBBP1A degradation and identification of ubiquitin ligases that degrade MYBBP1A may be a topic of interest for future studies.

We have very recently found that persistent depletion of rRNA processing factors, which mimics nucleolar status under replicative stress, leads to cellular senescence^46^. When rRNA processing was inhibited, nucleolar RNA content rather increased (Figs. 4h and S8e) and the nucleolus was enlarged (Figs. 4i,5a and S8e)^46^. Consistent with this result, MYBBP1A did not translocate from the nucleolus (Figs. 4i,5a and S8f). In this case, the nonacetylated form of p53 increased and p21 (but not BAX) accumulated (Figs. 4d,5b and S8a), which was independent of MYBBP1A but dependent on RPL11 (Figs. 4g and S8d). Depletion of almost all of the rRNA processing factors that we tested increased G1 phase cells without affecting the ratio of subG1 phase cells (Figs. 4e,5c and S8b), which would be a prerequisite for the induction of cellular senescence.

Post-translational modifications of p53, such as acetylation and phosphorylation, regulate sequence-specific DNA binding of p53^1^,^4^,^5^,^6^,^7^. Acetylation of p53 at the C-terminal region reportedly strengthens its DNA-binding activity^17^,^18^. Moreover, the strength of p53 DNA-binding activity determines promoter selectivity^16^,^55^,^56^. These observation suggest that nonacetylated p53 possesses weak DNA-binding activity and binds to REs on cell cycle genes, such as the *p21* gene, whereas acetylation of p53 at the C-terminal region confers enhanced DNA-binding activity, which leads to p53 binding to REs on proapoptotic targets, including *BAX*, *NOXA* and *PUMA*. Consistent with these observations, we showed that inhibiting rRNA transcription induced apoptosis via MYBBP1A by enhancing p53 K382-acetylation. To support this model, acetylated p53 robustly induced BAX expression (compare lane 5 with lanes 6 or 7; Fig. S11) even when p53 protein levels were similar, showing that p53 acetylation status influences BAX expression. However, as reported in the previous publication^26^, we still cannot exclude the effect of p53 accumulation on BAX expression from the result of Fig. 2c, in which BAX expression appears to be linked to protein levels of p53.

Finally, we propose that the nucleolus functions as a sensor that recognises the strength of cellular stress. Various types of stress, including anticancer drugs, gamma irradiation and glucose starvation, inhibit rRNA transcription^48^,^51^,^57^,^58^. These stressors inhibit rRNA transcription in a strength-dependent manner. Here we showed that MYBBP1A translocated from the nucleolus to the nucleoplasm in parallel with the inhibition of rRNA transcription (Figs. 1, S3, S5 and S6). Taken together, these results suggest that the nucleolus functions as a sensor that monitors the strength of cellular stress and induces MYBBP1A-dependent p53 acetylation and apoptosis by regulating the degree of rRNA transcription. Several compounds inhibit rRNA processing at a low dose but inhibit rRNA transcription at a high dose^51^, suggesting that lower stress inhibits rRNA processing and higher stress inhibits rRNA transcription. Thus, it is possible that the nucleolus induces cell cycle arrest and cellular senescence under lower stress conditions and induces apoptosis under higher stress conditions, at least in certain settings.

## Methods

### Cell culture & treatments

MCF-7 human breast cancer cells were maintained in Dulbecco’s modified Eagle’s medium containing 1000 mg/l glucose supplemented with 10% fetal bovine serum, 100 units/ml penicillin and 100 μg/ml streptomycin. Camptothecin (2 mg/ml; Sigma, St. Louis, MO, USA) treatment was generally performed 24 h after cell seeding.

### RNA interference

Cells at 30%–50% confluence were transfected with 20 nM siRNA using LipofectAmine RNAi max (Invitrogen, Carlsbad, CA, USA) to transfect the siRNAs, according to the manufacturer’s protocol, and were incubated for the indicated times. All siRNAs were purchased from Invitrogen. The siRNA sequences are shown in Supplementary Table S1. The Stealth RNAi^TM^ Luciferase reporter control duplex was used as a negative control.

### Antibodies

The following antibodies were used in these experiments: β-actin (Sigma), p53 (DO-1, Santa Cruz Biotechnology, Santa Cruz, CA, USA), acethyl-p53 (lysine382, Cell Signaling Technology, Danvers, MA, USA), p21 (F-5, Santa Cruz Biotechnology), BAX (Abcam, Cambridge, MA, USA), PARP (Cell Signaling Technology), Cyclin A (H-432, Santa Cruz Biotechnology), Cyclin D1 (Cell Signaling Technology), POLR1B (N-17, Santa Cruz Biotechnology), UBF (F-9, Santa Cruz Biotechnology), POLR1A (C-1, Santa Cruz Biotechnology), TIF-IA (C-20, Santa Cruz Biotechnology), PES1 (Bethyl Laboratories, Montgomery, TX, USA), WDR3 (Acris Antibodies, San Diego, CA, USA), NOL1 (Bethyl Laboratories), UTP6 (Gene Tex, Irvine, CA, USA) and RPL11 (3A4A7, Invitrogen). The rabbit anti-MYBBP1A antibody was raised against a synthetic peptide corresponding to the human-MYBBP1A 1265–1328 amino acid sequence.

### Immunofluorescent staining

Immunofluorescent staining was performed as described previously^48^. MCF-7 cells were fixed in 4% paraformaldehyde at room temperature for 15 min and permeabilized with 0.5% Triton X-100 buffer (20 mM HEPES, 150 mM KCl and 0.5% Triton X-100) for 10 min at room temperature. Samples were blocked with 0.5% goat serum in TBS-T buffer (20 mM Tris pH 7.5, 150 mM NaCl and 0.05% Tween-20) for 1 h, washed with phosphate-buffered saline (PBS; 140 mM NaCl, 2.7 mM KCl, 1.5 mM KH_2_PO_4_ and 8.1 mM Na_2_HPO_4_), and incubated with the indicated antibodies for 2 h. The samples were washed with PBS and incubated with Alexa Fluor^®^ 594 goat anti-mouse IgG (H + L) (Invitrogen) or Alexa Fluor^®^ 488 goat anti-rabbit IgG (H + L) (Invitrogen) for 1 h. The samples were washed again with PBS, stained with 4, 6-diamidino-2-phenylindole (Dojindo, Rockville, MD, USA), and mounted on slides with Vectashield (Vector Laboratories, Burlingame, CA, USA). The samples were visualized by Biozero immunofluorescence microscopy (Keyence, Osaka, Japan).

### Immunoblotting

Cells were harvested by trypsinization, washed with PBS, and lysed in RIPA buffer [25 mM Tris-HCl, pH 7.6, 150 mM NaCl, 1% NP-40, 1% Triton X-100, 1% sodium deoxycholate, and 0.1% sodium dodecyl sulfate-polyacrylamide (SDS) plus a protease inhibitor cocktail (Nacalai Tesque, Inc., Kyoto, Japan) for 30 min on ice. The lysates were cleared by centrifugation at 16,100 × *g* for 30 min at 4 °C. Protein concentrations were evaluated using a BCA kit (Thermo Scientific, Rockford, IL, USA). Extracted proteins were separated by SDS-polyacrylamide gel electrophoresis and transferred to polyvinylidene fluoride membranes (Millipore, Milford, MA, USA). After blocking with 5% skim milk in TBS-T buffer- for 1 h, the membranes were incubated with primary antibodies for at least 1 h. After washing with TBS-T buffer 3 times, the membranes were incubated with horseradish peroxidase-conjugated secondary antibody for 1 h. Then the membrane was washed with TBS-T buffer twice and with TBS once. Bands were detected with Chemi-Lumi One (Nacalai Tesque) or the Immobilon Western blotting detection kit (Millipore).

### Nucleoli purification and quantitative determination of nucleolar RNA content

Nucleoli were isolated from 1.2 × 10^7^ MCF-7 cells by density gradient fractionation, as described previously^59^. Total nucleolar RNA was prepared from the isolated nucleoli and quantified by spectrometry.

### Northern blotting

RNA was isolated with a FastPure^®^ RNA kit (Takara Bio) according to the manufacturer’s instructions. Total RNA (2 μg) was separated on a 1% agarose-formaldehyde gel and blotted on nylon membranes (GE Healthcare, Bucks, UK). The following DNA oligonucleotides were 5`end-labeled using T4 polynucleotide kinase in the presence of [*g*-^32^P] ATP: 5′-CCTCCGCGCCGGAACGCGCTAGGTACCTGGACGGCGGGGGGGCGGACG-3′ for ITS1. Membranes were preincubated with ULTRAhyb buffer (Ambion, Austin, TX, USA) at 65 °C for 30 min, and the radioactively labeled probes were then added overnight. Membranes were shortly washed in 1 × SSC and 0.2 × SSC.

### RNA purification and reverse transcription-quantitative polymerase chain reaction (RT-qPCR)

Total RNA was isolated with a FastPure^®^ RNA kit (Takara Bio, Shiga, Japan) according to the manufacturer’s instructions. Total RNA (1 μg) was reverse transcribed with the PrimeScript^®^ 1^st^ Strand cDNA Synthesis kit (Takara Bio). The real-time quantitative PCR analysis was performed to amplify fragments representing the indicated mRNA or for pre-rRNA expression using the Thermal Cycle Dice^TM^ TP800 (Takara) and SYBR. Indicated mRNA or pre-rRNA levels were normalised to β-actin mRNA levels. The primer sequences can be found in Supplementary Table S2.

### ChIP assay

ChIP assay was performed as described previously^60^ with minor modifications. DNA was amplified by RT-qPCR as described above. The primers for real-time PCR were p53-binding site in the human *p21* gene promoter region forward primer: 5′-GTGGCTCTGATTGGCTTTCTG-3′, human *p21* gene reverse primer: 5′-CTTGGGCTGCCTGTTTTCAG-3′; human *BAX* gene forward primer: 5′-TAATCCCAGCGCTTTGGAAG-3′; and the human *BAX* gene reverse primer: 5′-TTGCTAGATCCAGGTCTCTGCA-3′. Samples were normalised based on the amount of inputted DNA.

### Cell cycle analysis

The cell cycle was analysed as described previously^48^. The cells were detached with trypsin, collected with the supernatant, and washed twice with ice-cold PBS. The cells were fixed in 75% (v/v) cold ethanol for no less than 12 h and washed in ice-cold PBS. After removing the supernatant, the cells were stained with the Guava^®^ Cell Cycle Reagent (Millipore). Cell cycle populations were determined using the Guava^®^ EasyCyte system (Guava Technologies, Hayward, CA, USA), according to the manufacturer’s recommendations. Guava^®^ Cell Cycle software (Guava Technologies) was used to determine the different cell cycle phases in the cell populations.

## Additional Information

**How to cite this article**: Kumazawa, T. *et al.* Gradual reduction in rRNA transcription triggers p53 acetylation and apoptosis via MYBBP1A. *Sci. Rep.* **5**, 10854; doi: 10.1038/srep10854 (2015).

## Supplementary Material

Supplementary Information
