# Supplementary material for: Gradual reduction in rRNA transcription triggers p53 acetylation and apoptosis via MYBBP1A
Source: Sci Rep. 2015 Jun 5;5:10854. doi: 10.1038/srep10854 (PMC4456663; doi:10.1038/srep10854)
Supplement: Supplementary Information [file srep10854-s1.pdf]

Gradual reduction in rRNA transcription triggers p53 acetylation and apoptosis via MYBBP1A

Takuya Kumazawa, Kazuho Nishimura, Naohiro Katagiri, Sayaka Hashimoto, Yuki Hayashi and Keiji Kimura

1. Supplementary Tables

Table S1, related to Methods

Table S2, related to Methods

2. Supplemental Figures

Figure S1, related to Figure 1f

Figure S2, related to Figure 1

Figure S3, related to Figure 2

Figure S4, related to Figure 3g

Figure S5, related to Figure 3

Figure S6, related to Figure 3

Figure S7, related to Figure 4e

Figure S8, related to Figure 4

Figure S9, related to Figure 5c

Figure S10, related to Discussion

Figure S11, related to Discussion

Figure S12, complete scan of the blots presented in Figure 1b

Figure S13, complete scan of the blots presented in Figure 1e

Figure S14, complete scan of the blots presented in Figure 1g

Figure S15, complete scan of the blots presented in Figure 2c

Figure S16, complete scan of the blots presented in Figure 3b

Figure S17, complete scan of the blots presented in Figure 3f

Figure S18, complete scan of the blots presented in Figure 3h

Figure S19, complete scan of the blots presented in Figure 4b

Figure S20, complete scan of the blots presented in Figure 4d

Figure S21, complete scan of the blots presented in Figure 4f

Figure S22, complete scan of the blots presented in Figure 4g

Figure S23, complete scan of the blots presented in Figure 5b

Figure S24, complete scan of the blots presented in Figure 5d

Supplementary Table S1. Target sequences used in siRNA experiments, related to Methods

Target	Sequence (5’→3’)
POLR1B	GAGCAAGCUGCGGAGUUCCUGUUUA
UBF	UUCGGCUGCCUUCUUAAUCCACAUC
POLR1A	CCUAGGAGACCAGAUGUUUACUAAU
TIF-IA#1	CGACACCGUGGUUUCUCAUGCCAAU
TIF-IA#2	AGGAUGUCUGCUAUGUAGAUGGUAA
PES1	CCAUUGUCAACAAGUUCCGUGAAUA
WDR3	GGGACAUAGCUUAUCUGCAAGAGAU
NOL1	GGUCAAAGACUGGACUAGUGGUGUA
UTP6	GCUAUGCAUCAGACUGGCCAUUUAU
EMG1	UAGGGAAGACAUAUGAGCUACUCAA
SKIV2L2	GGGAAUUAACAUGCCAGCUAGAACU
FCF1	CAAGGAUCCAAGAUUUGAACGAUUA
BMS1	CAGUGCAGAGGAAGAAGACUCAGAA
UTP3	AUAAUUCACCACUAUAACGUUGCUC
NOL7	CCAGGAAAGGUGAAAGAAGUUAAUU
WDR46	UCCAGAAGUUCUGUCGCAUUGACAA
UTP11L	GGAGUUACCAAUCAGACUGGACUUA
NOL6	CCACCUGCUGUUGAUGACUCCCAAA
NOL14	AUCAAUGACUGUGAGGUCUGGUGGG
p53	CCAGUGGUAAUCUACUGGGACGGAA
RPL11	GCUAGAUACACUGUCAGAUCCUUUG
MYBBP1A	UCUUUCAGUCAGGUCGGCUGGUGAA

Supplementary Table S2. Primer sequences used for reverse transcription-quantitative reverse transcription-polymerase chain reaction (RT-qPCR), related to Methods

	Forward	Reverse
pre-rRNA	GAACGGTGGTGTGTCGTTC	GCGTCTCGTCTCGTCTCACT
POLR1B	GGAATCTGCGTTCTAAAACAGG	TGGGAGAGGTAGCGTATGAAG
UBF	GGAGACATGTGCAAGCTCAA	GAGCATCGAGGATCAATTCTGTC
POLR1A	CAACTGTGAACACGATGCAGA	TCCGACCTTCCAGTTCAATC
TIF-IA	CCATGAAAAAGGACATAGTG	CGTGTCAAAGGAGCTTGGT
PES1	TGCAGACCATTCAGCTGTGC	GCATAGGGAGTGATCCACAC
WDR3	TATGATCAGCTAGGAGGCAG	CTTTCCCACTAGTAACTAGC
NOL1	GAAATTGGGGGACGAAGAGT	GCAGGCAAGAATCTGACGAG
UTP6	TCAGGAACGCATAGAAGATCG	AGATCGGAAGCCTTCTTAATGAT
β-actin	CCAACCGCGAGAAGATGA	CCAGAGGCGTACAGGGATAG

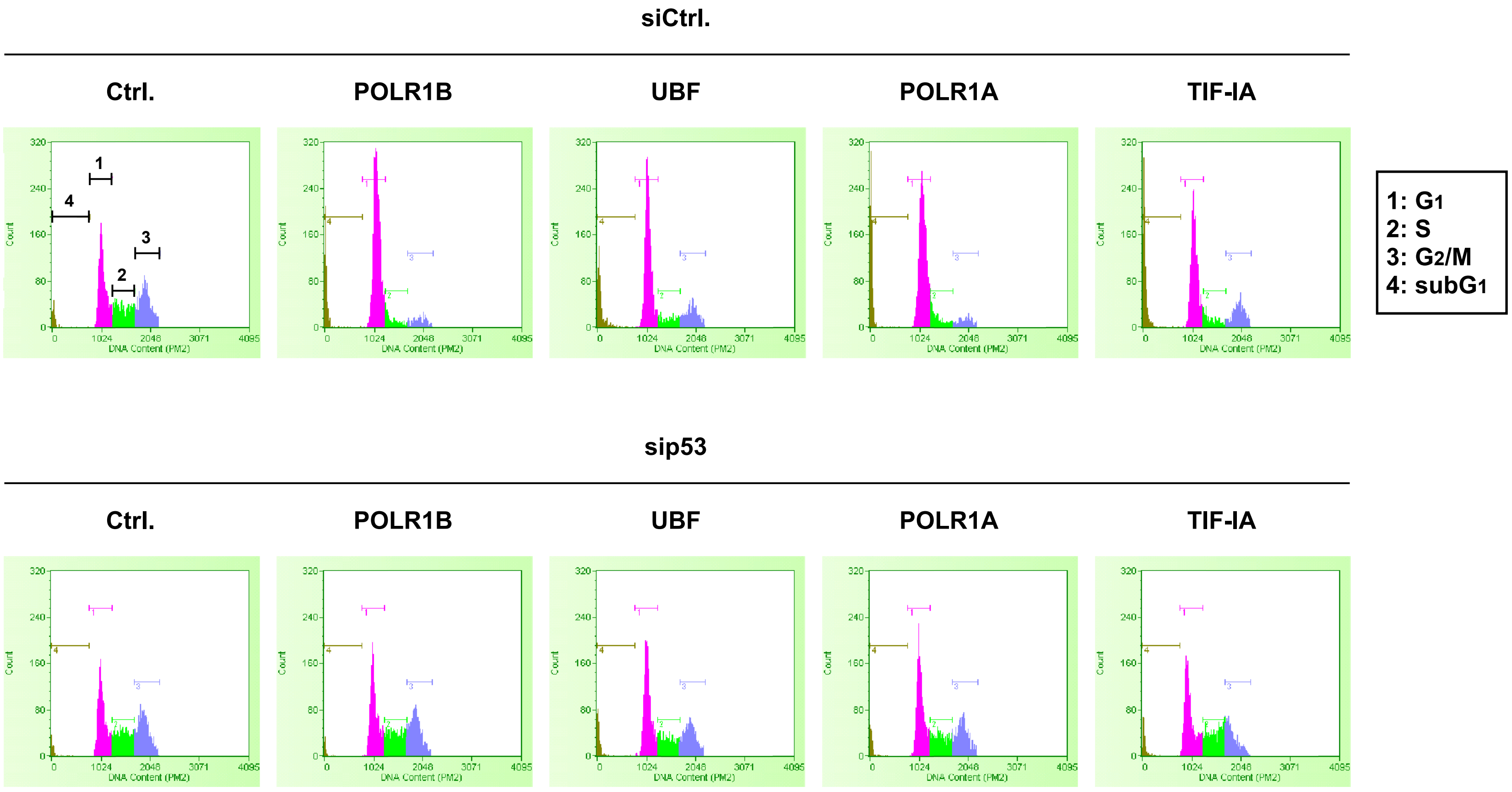

Supplementary Figure S1. Inhibition of rRNA transcription causes G1 cell cycle arrest and apoptosis in MCF-7 cells
An example of FACS profiles of Figure 1f are shown. 1, G1 phase; 2, S phase; 3, G2/M phase; 4, subG1 phase

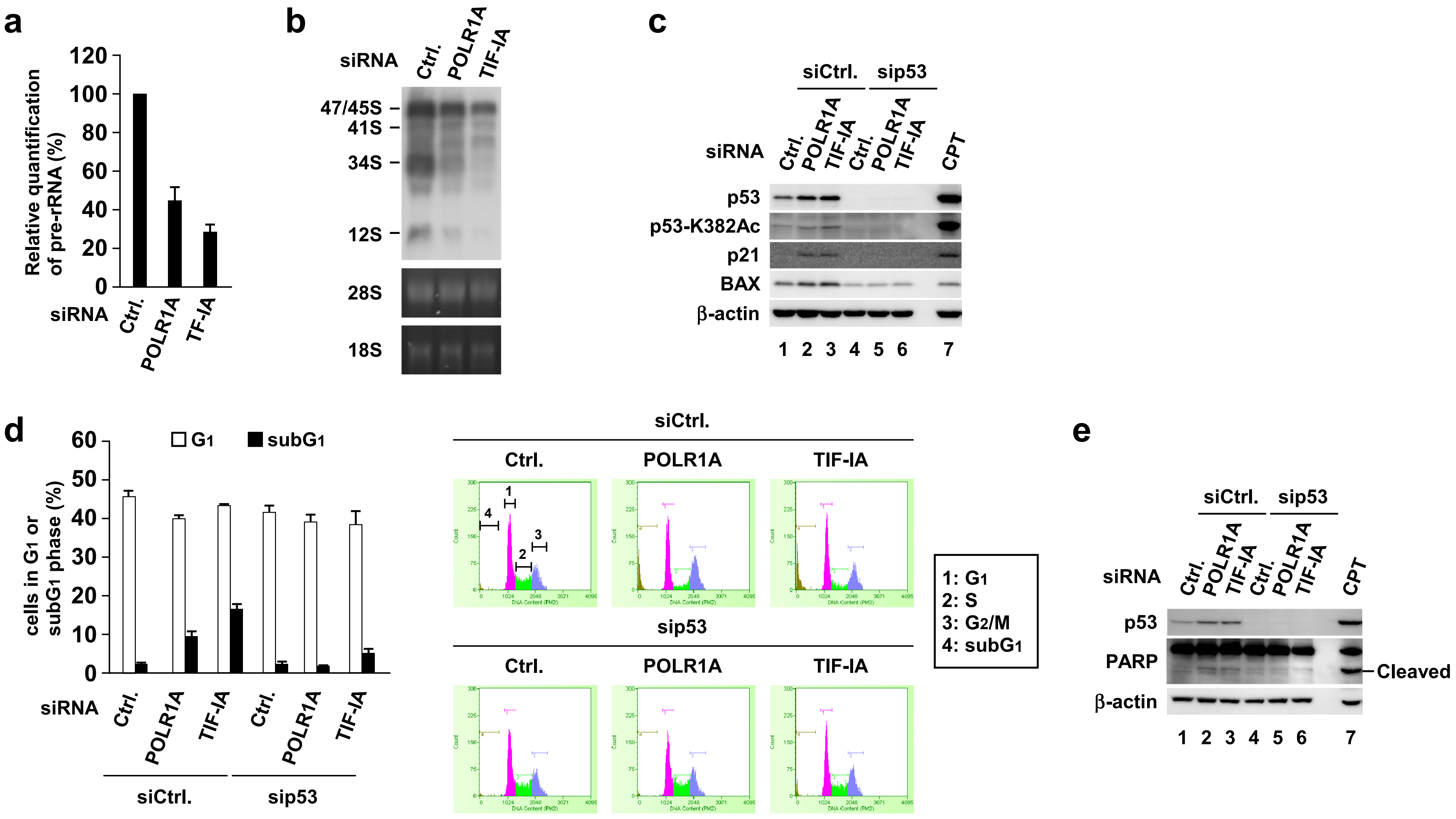

Supplementary Figure S2. Reduction in pre-rRNA levels correlates with p53 activation and apoptosis induction in U2OS cells

(a) U2OS cells were transfected with siCtrl, siPOLR1A, or siTIF-IA for 60 h. pre-rRNA transcription was assessed by RT-qPCR (n = 3). Error bars indicate mean ± standard deviation (SD). (b) U2OS cells were transfected with the indicated siRNAs for 60 h. Northern blotting was performed using a probe specific for the rRNA internal transcribed spacer 1 region. An ethidium bromide-stained gel is shown at the bottom as a loading control. (c-e) U2OS cells were transfected with combinations of siRNAs as indicated. (c) Cell lysates were prepared at 60 h after transfection and immunoblotted using the indicated antibodies. Lysates from 2 µg/ml camptothecin (CPT)-treated cells were used as a positive control. (d) DNA content was determined by flow cytometry at 84 h after transfection (n = 3). Error bars indicate mean ± SD. An example of FACS profiles are shown on the right. (e) Cell lysates were prepared at 84 h after transfection and immunoblotted using the indicated antibodies. Lysates from 2 µg/ml CPT-treated cells were used as a positive control.

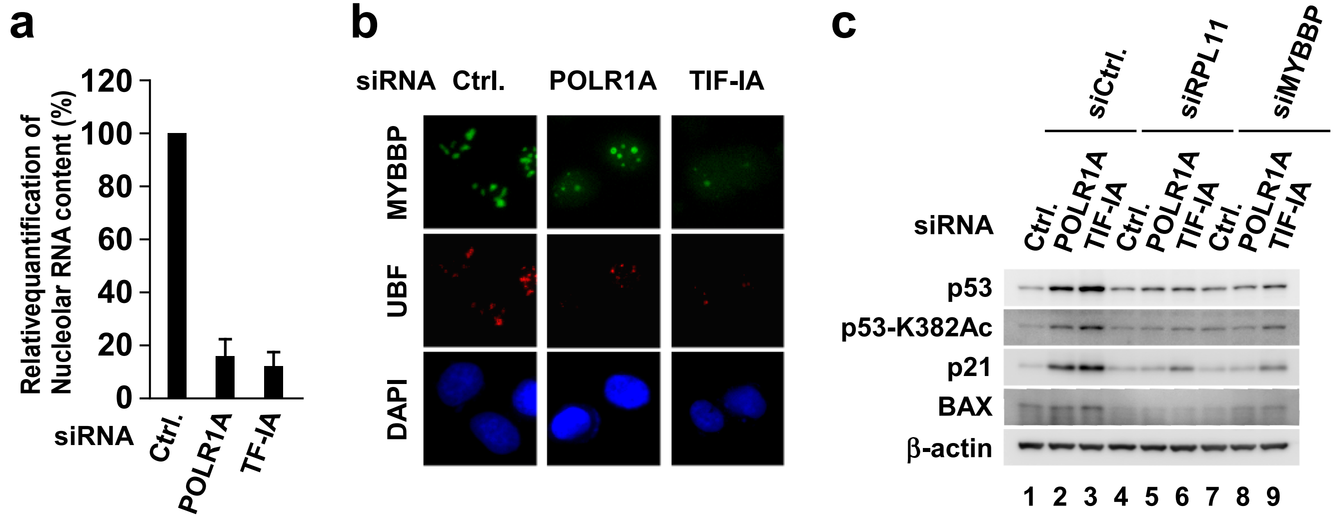

Supplementary Figure S3. Reduction in nucleolar RNA content induces MYBBP1A translocation and p53 activation in U2OS cells

(a) U2OS cells were transfected with siCtrl, siPOLR1A or siTIF-IA. Nucleolar RNA content was spectrophotometrically quantified at 60 h after transfection (n = 3). Nucleolar RNA content of control cells was normalised to 100%. Error bars indicate mean ± standard deviation. (b) U2OS cells were transfected with the indicated siRNAs. Immunofluorescence staining was performed using the indicated antibodies at 60 h after transfection. (c) U2OS cells were transfected with combinations of siRNAs as indicated for 60 h. Cell lysates were prepared from these cells and were immunoblotted using the indicated antibodies.

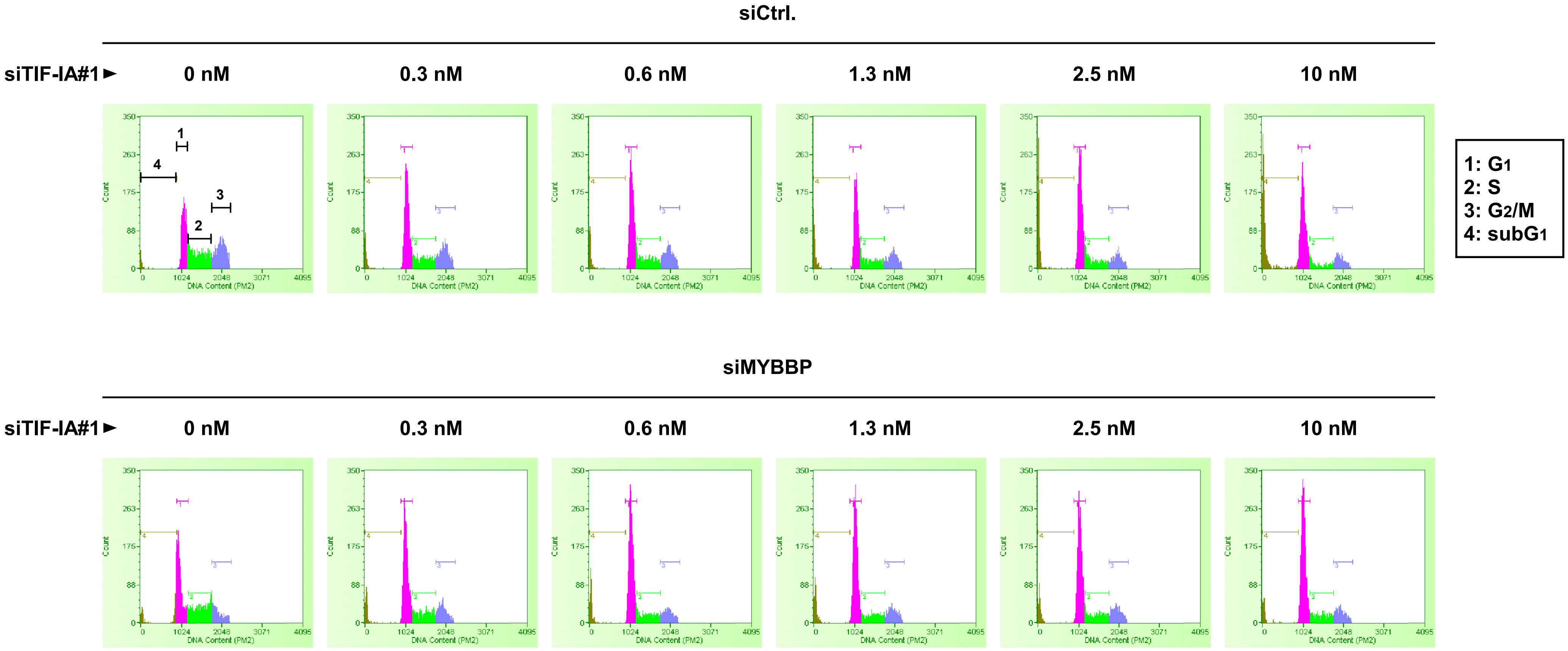

Supplementary Figure S4. siTIF-IA#1 treatment gradually induces MYBBP1A-dependent apoptosis in a dose-dependent manner
An example of FACS profiles of Figure 3g are shown. 1, G1 phase; 2, S phase; 3, G2/M phase; 4, subG1 phase

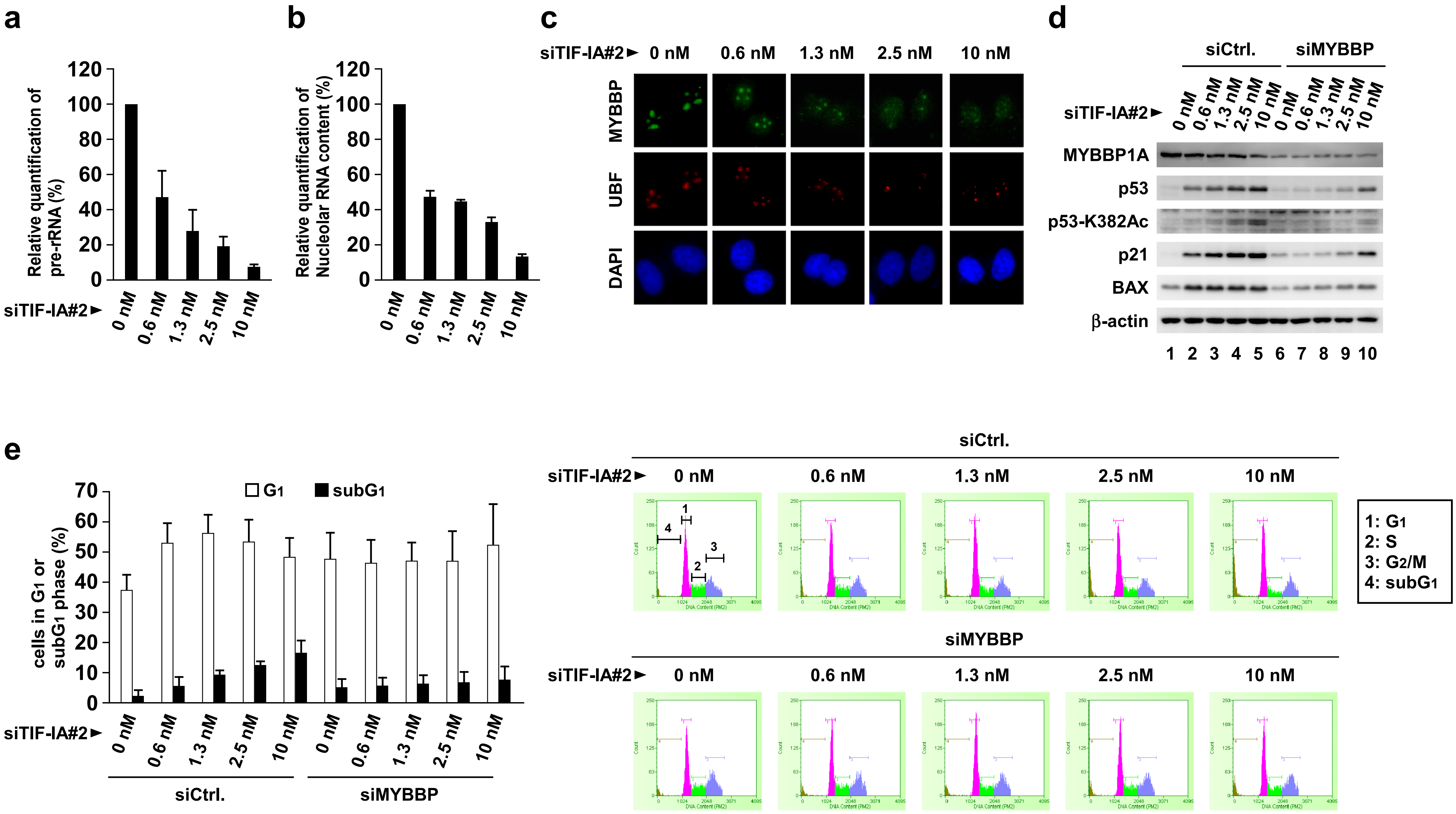

Supplementary Figure S5. siTIF-IA#2 treatment dose-dependently causes MYBBP1A-dependent p53 activation and apoptosis in MCF-7 cells
(a-c) MCF-7 cells were transfected with the indicated concentrations of siTIF-IA#2. (a) pre-rRNA transcription was assessed by RT-qPCR at 48 h after transfection (n = 3). Error bars indicate mean ± standard deviation (SD). (b) Nucleolar RNA was isolated from purified nucleoli and quantified by spectrophotometry at 60 h after transfection (n = 3). Nucleolar RNA content of control cells was normalised to 100%. Error bars indicate mean ± SD. (c) Immunofluorescence staining was performed using the indicated antibodies at 60 h after transfection. (d and e) MCF-7 cells were transfected with combinations of siRNAs as indicated. (d) Cell lysates were prepared at 60 h after transfection and were immunoblotted using the indicated antibodies. (e) DNA content was determined by flow cytometry at 84 h after transfection (n = 3). Error bars indicate mean ± SD. An example of FACS profiles are shown on the right.

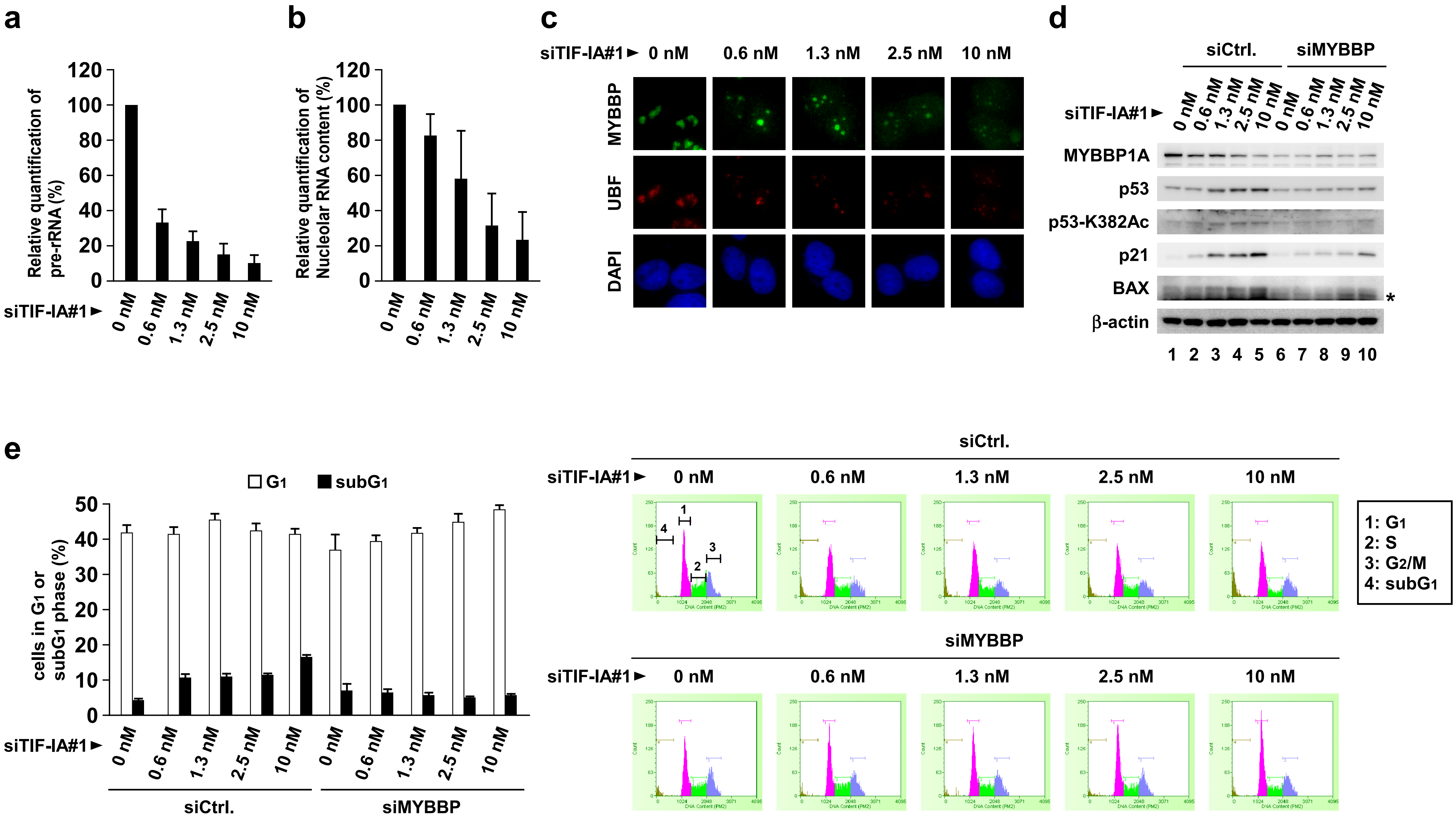

Supplementary Figure S6. siTIF-IA#1 treatment dose-dependently causes MYBBP1A-dependent p53 activation and apoptosis in U2OS cells
(a-c) U2OS cells were transfected with the indicated concentrations of siTIF-IA#1. (a) pre-rRNA transcription was assessed by RT-qPCR at 48 h after transfection (n = 3). Error bars indicate mean ± standard deviation (SD). (b) Nucleolar RNA was isolated from purified nucleoli and quantified by spectrophotometry at 60 h after transfection (n = 3). Nucleolar RNA content of control cells was normalised to 100%. Error bars indicate mean ± SD. (c) Immunofluorescence staining was performed using the indicated antibodies at 60 h after transfection. (d and e) U2OS cells were transfected with combinations of siRNAs as indicated. (d) Cell lysates were prepared at 60 h after transfection and were immunoblotted using the indicated antibodies. Asterisk indicates nonspecific bands. (e) DNA content was determined by flow cytometry at 84 h after transfection (n = 3). Error bars indicate mean ± SD. An example of FACS profiles are shown on the right.

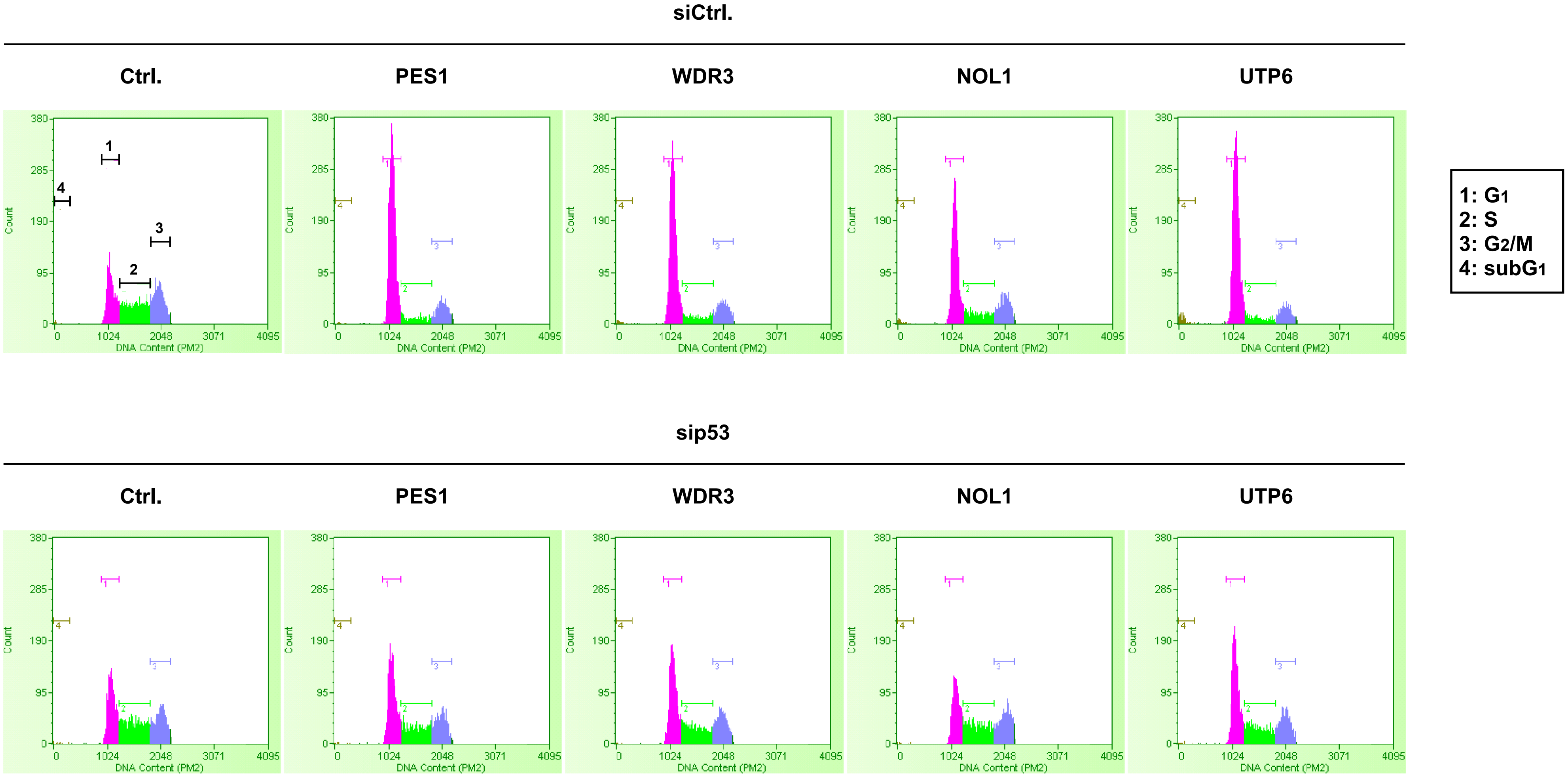

Supplementary Figure S7. Depleting rRNA processing factors causes G1 cell cycle arrest but not apoptosis in MCF-7 cells
An example of FACS profiles of Figure 4e are shown. 1, G1 phase; 2, S phase; 3, G2/M phase; 4, subG1 phase

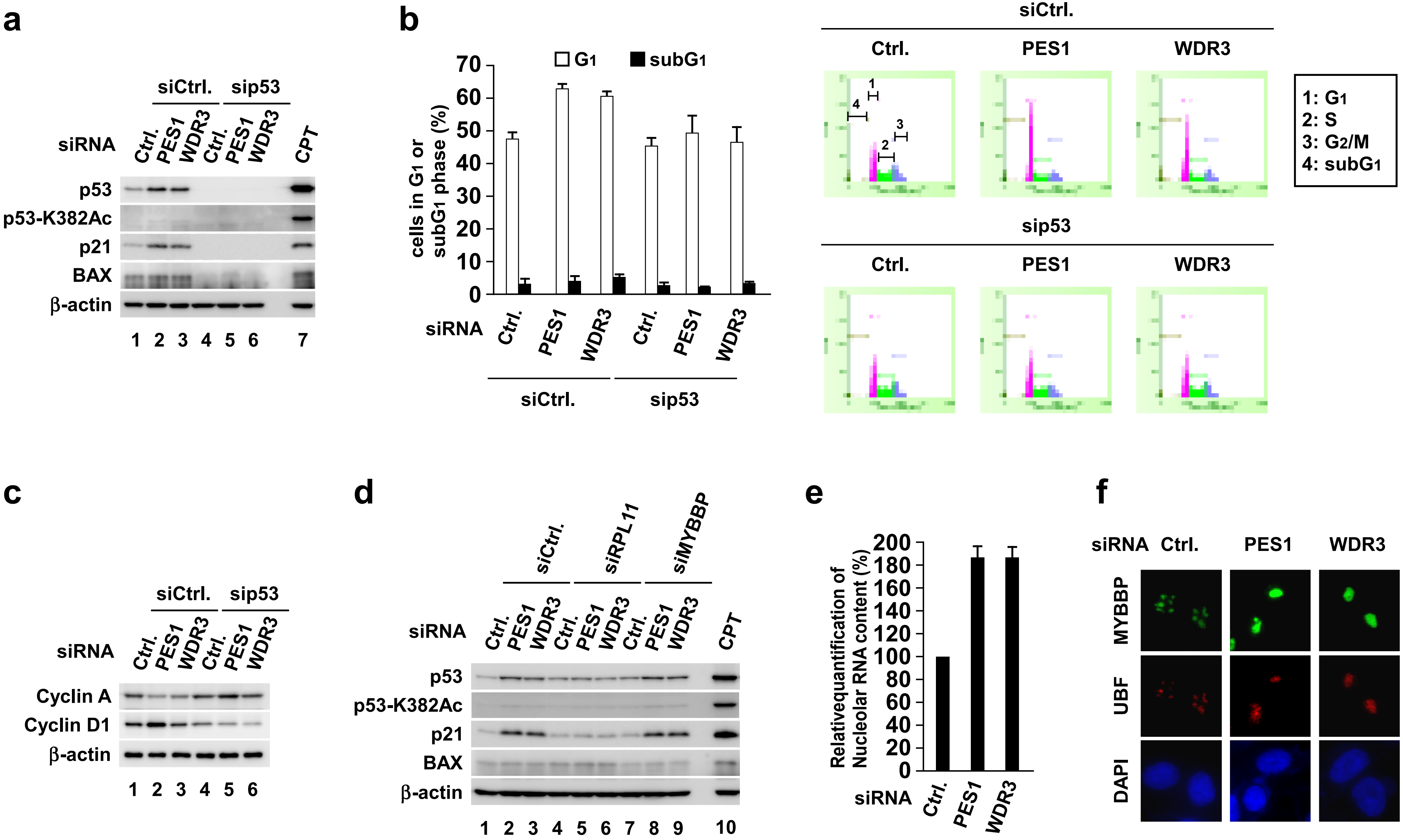

Supplementary Figure S8. Depleting rRNA processing factors causes RPL11-dependent and MYBBP1A-independent p53 activation in U2OS cells
(a-c) U2OS cells were transfected with combinations of siRNAs as indicated. (a) Cell lysates were prepared at 60 h after transfection and immunoblotted using the indicated antibodies. Lysates from 2 µg/ml camptothecin (CPT)-treated cells were used as the positive control. (b) DNA content was determined by flow cytometry at 84 h after transfection (n = 3). Error bars indicate mean ± standard deviation (SD). An example of FACS profiles are shown on the right. (c) Cell lysates were prepared at 72 h after transfection and immunoblotted using the indicated antibodies. (d) Cell lysates were prepared at 60 h after transfection and immunoblotted using the indicated antibodies. Lysates from 2 µg/ml CPT-treated cells were used as the positive control. (e) Nucleolar RNA content was spectrophotometrically quantified at 60 h after transfection (n = 3). Nucleolar RNA content of control cells was normalised to 100%. Error bars indicate mean ± SD. (f) Immunofluorescence staining was performed using the indicated antibodies at 60 h after transfection.

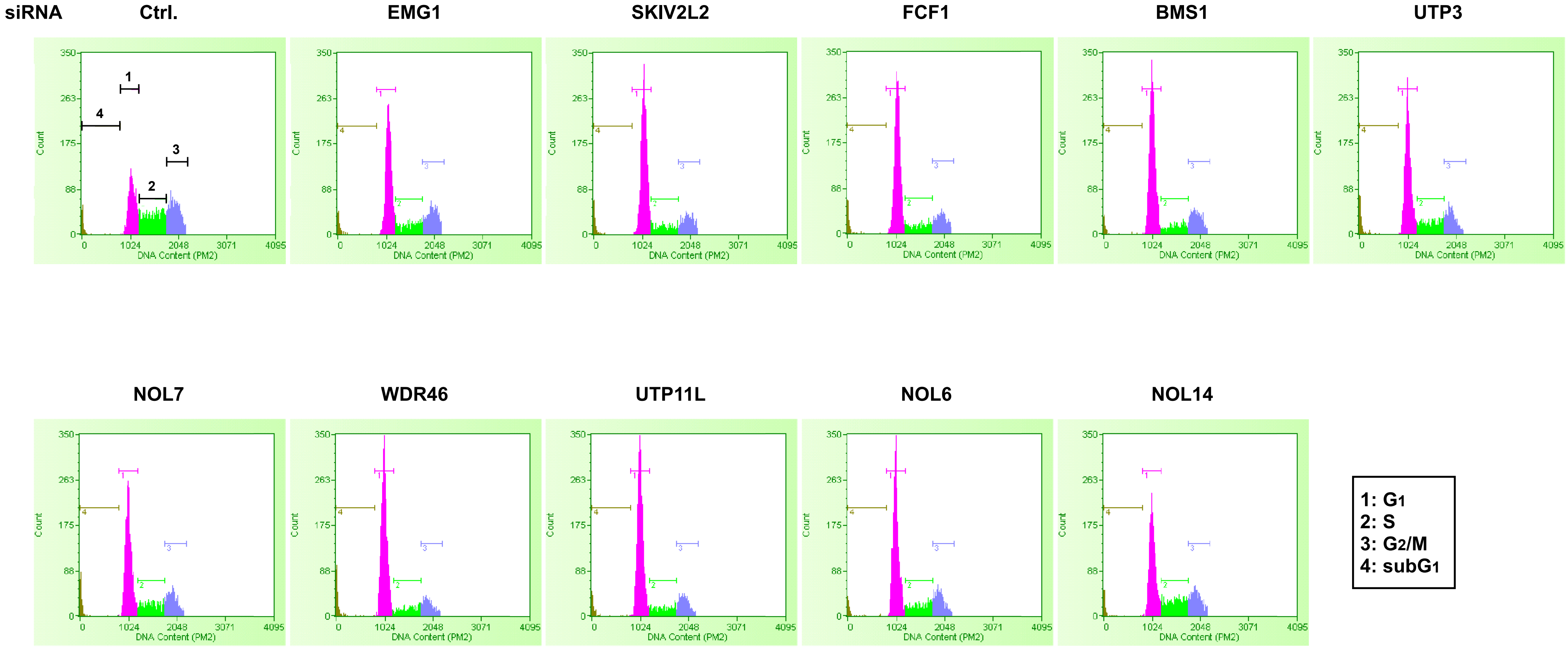

Supplementary Figure S9. Depletion of various rRNA processing factors causes G1 cell cycle arrest but not apoptosis in MCF-7 cells

An example of FACS profiles of Figure 5c are shown. 1, G1 phase; 2, S phase; 3, G2/M phase; 4, subG1 phase

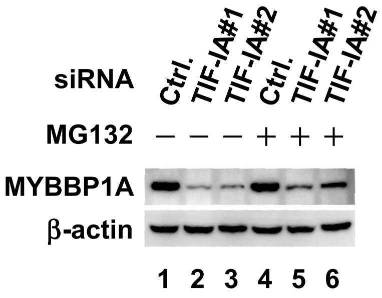

Supplementary Figure S10. MG132 alleviates the reduction in MYBBP1A protein levels by siTIF-IA treatment

MCF-7 cells were transfected with the indicated siRNAs. At 60 h after siRNA treatment, 0 or 10 μM MG132 was added to the culture medium and cultured for another 10 h. Next, cell lysates were analysed by immunoblotting with the indicated antibodies.

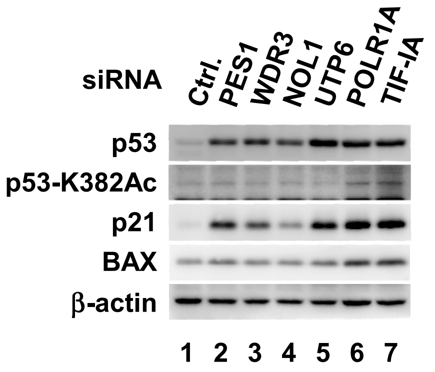

Supplementary Figure S11. p53 acetylation and BAX expression is robustly induced by depletion of rRNA transcription factors

MCF-7 cells were transfected with siRNAs against rRNA processing factors (PES1, WDR3, NOL1 and UTP6) or rRNA transcription factors (POLR1A and TIF-IA) for 60 h. Cell lysates were prepared from these cells and immunoblotted using the indicated antibodies.

Fig. 1b

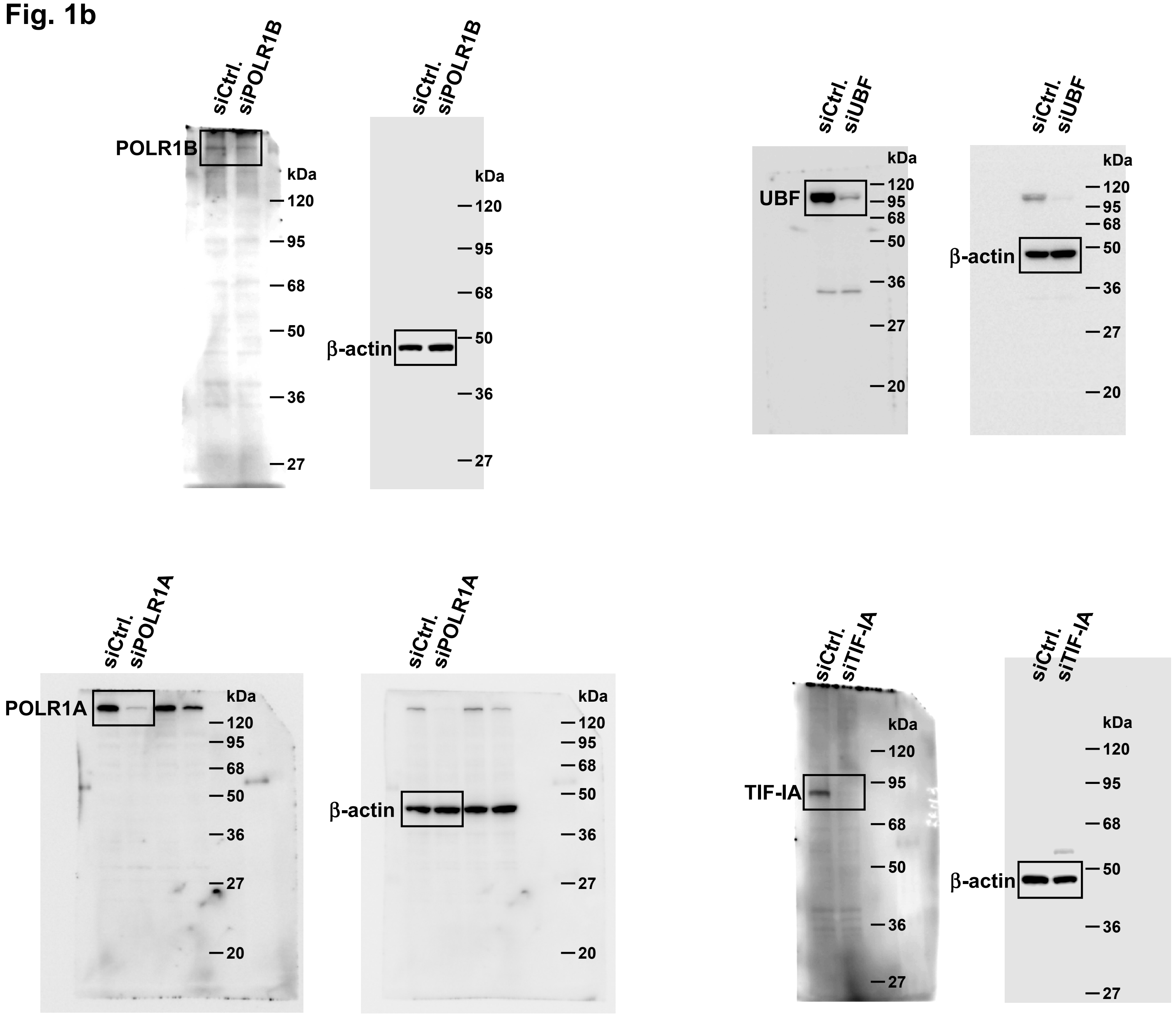

Supplementary Figure S12. complete scan of the blots presented in Figure 1b

Fig. 1e

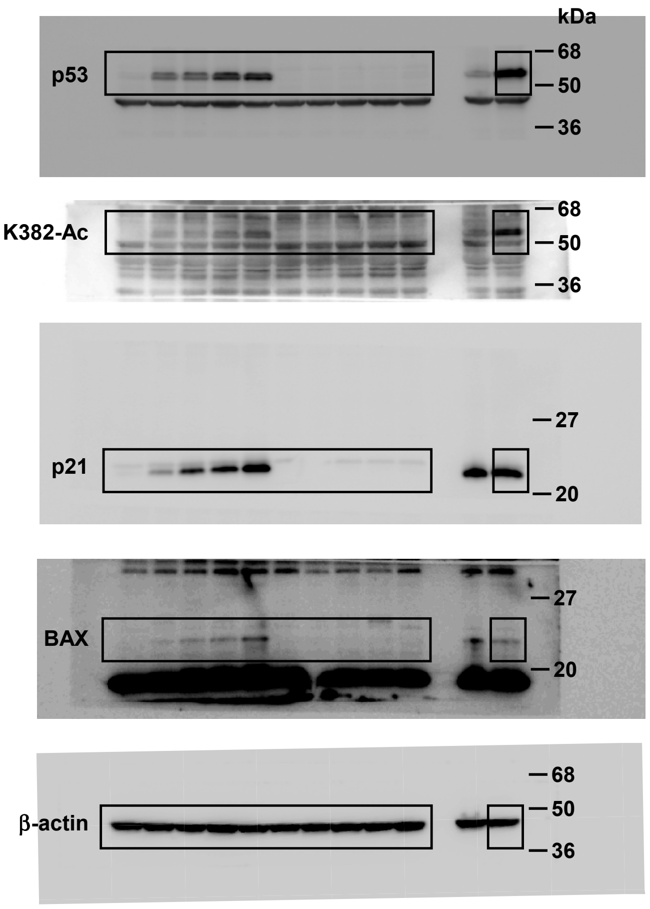

Supplementary Figure S13. complete scan of the blots presented in Figure 1e

Fig. 1g

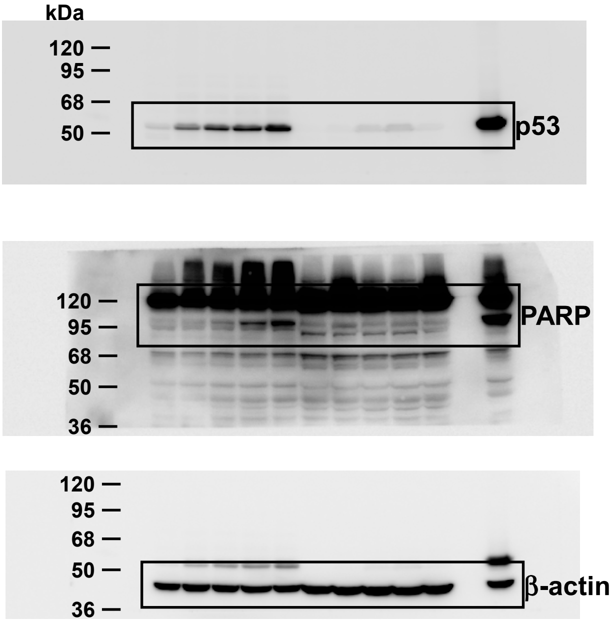

Supplementary Figure S14. complete scan of the blots presented in Figure 1g

Fig. 2c

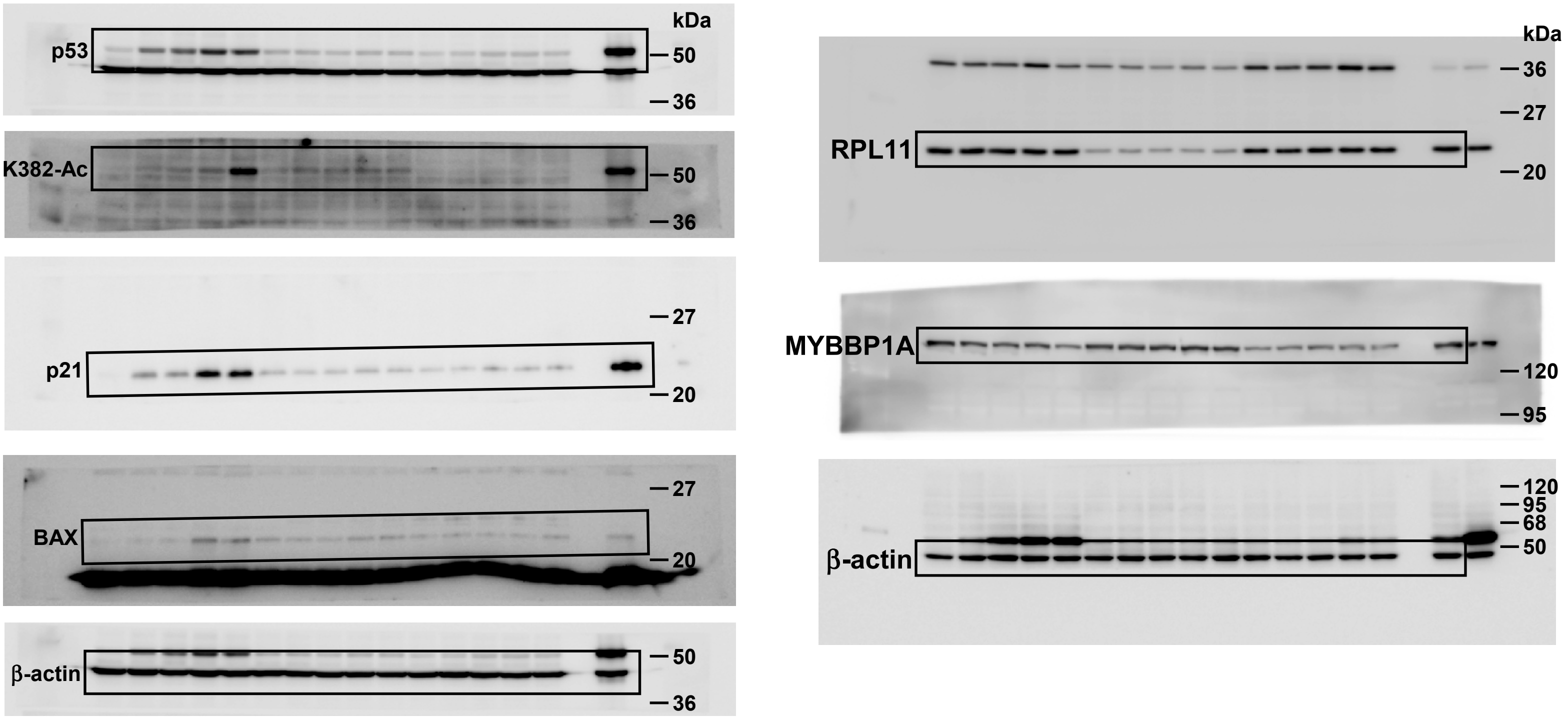

Supplementary Figure S15. complete scan of the blots presented in Figure 2c

Fig. 3b

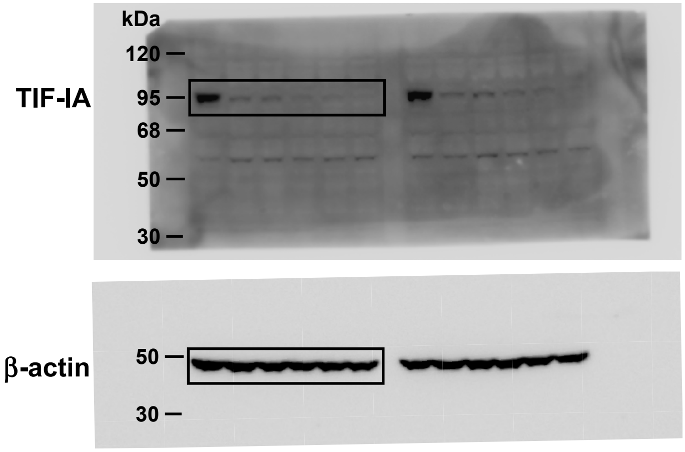

Supplementary Figure S16. complete scan of the blots presented in Figure 3b

Fig. 3f

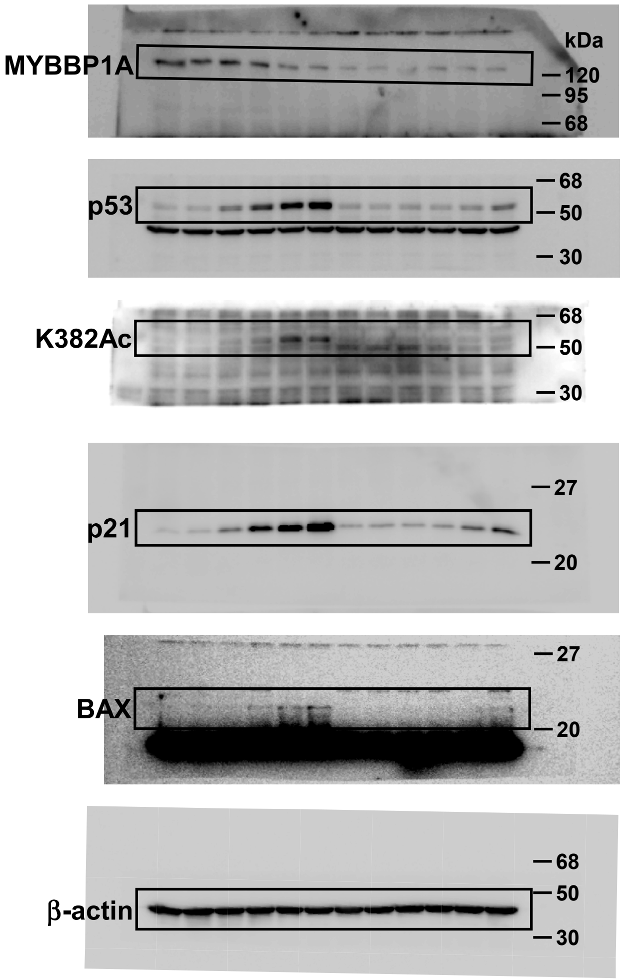

Supplementary Figure S17. complete scan of the blots presented in Figure 3f

Fig. 3h

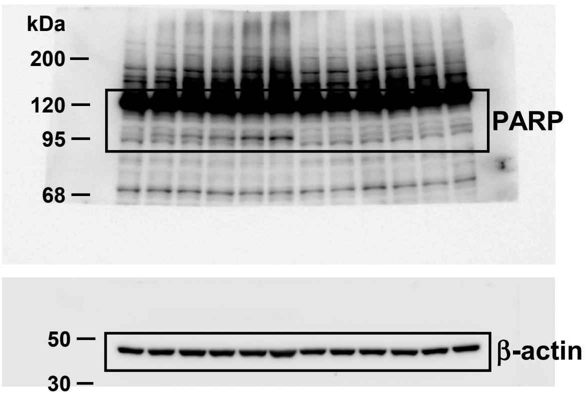

Supplementary Figure S18. complete scan of the blots presented in Figure 3h

Fig. 4b

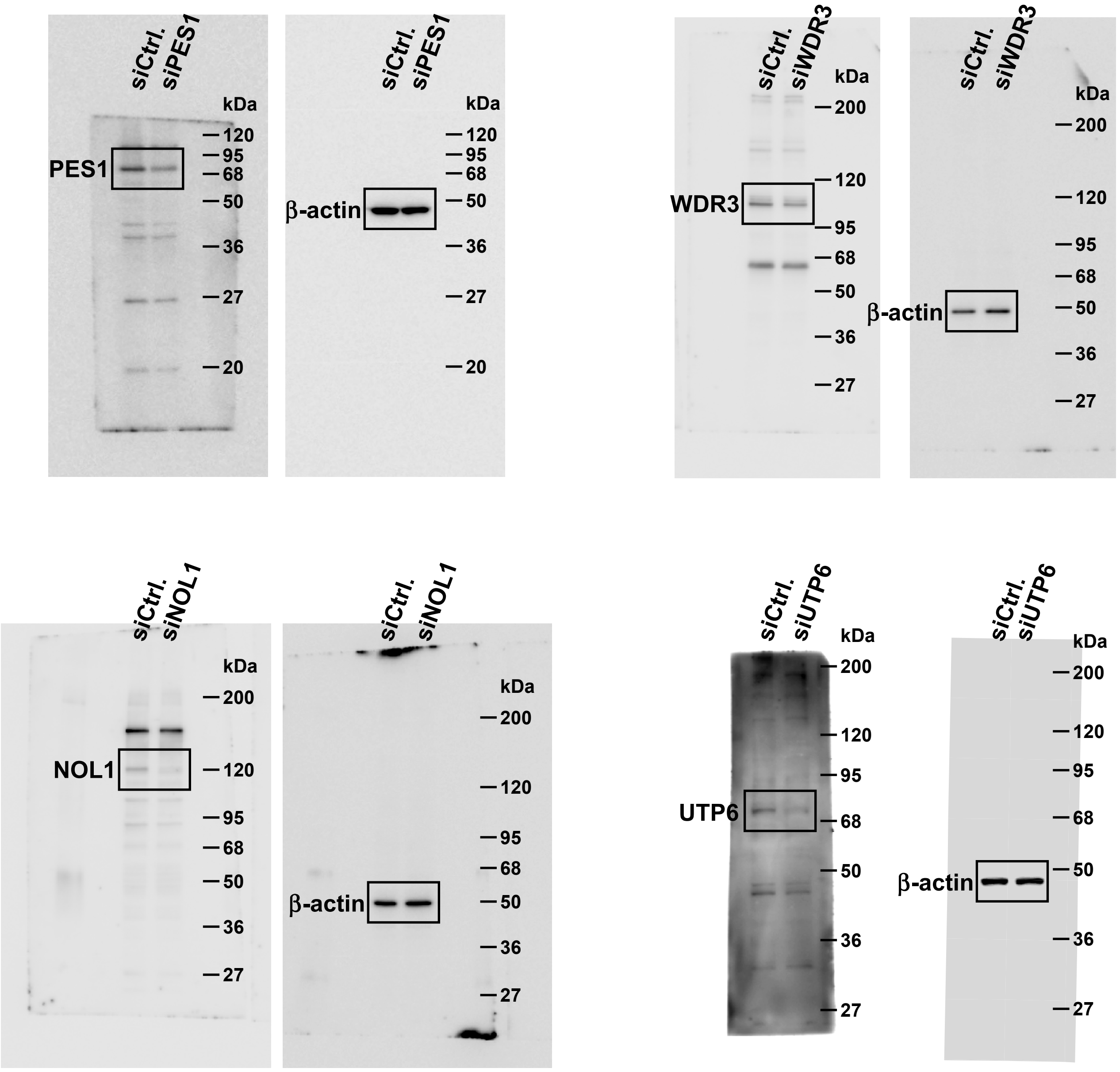

Supplementary Figure S19. complete scan of the blots presented in Figure 4b

Fig. 4d

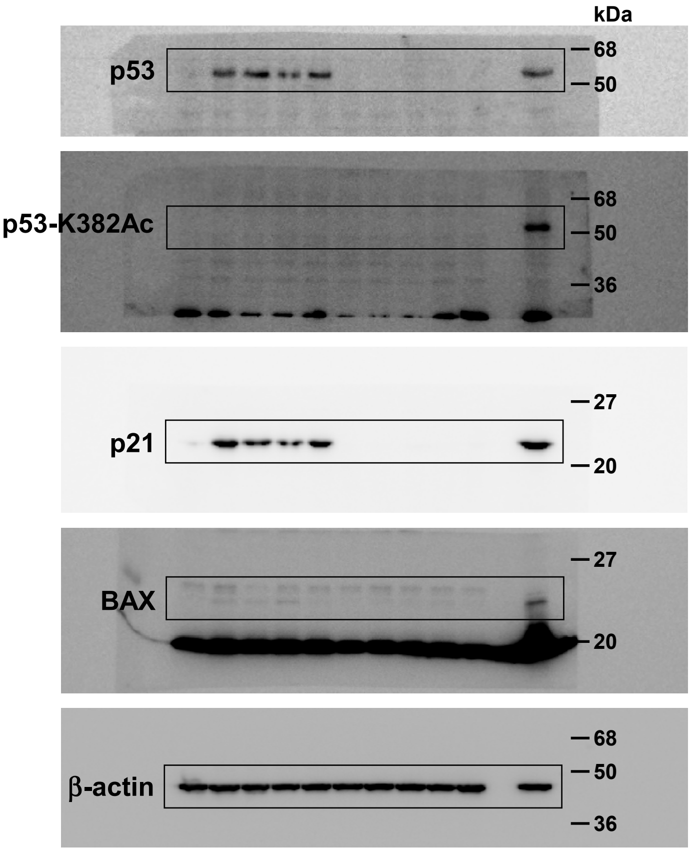

Supplementary Figure S20. complete scan of the blots presented in Figure 4d

Fig. 4f

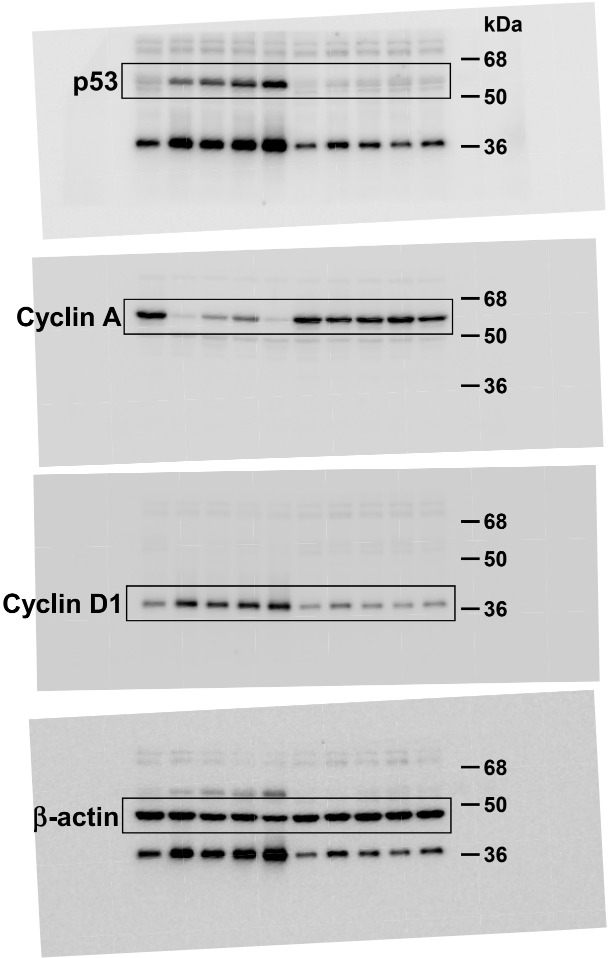

Supplementary Figure S21. complete scan of the blots presented in Figure 4f

Fig. 4g

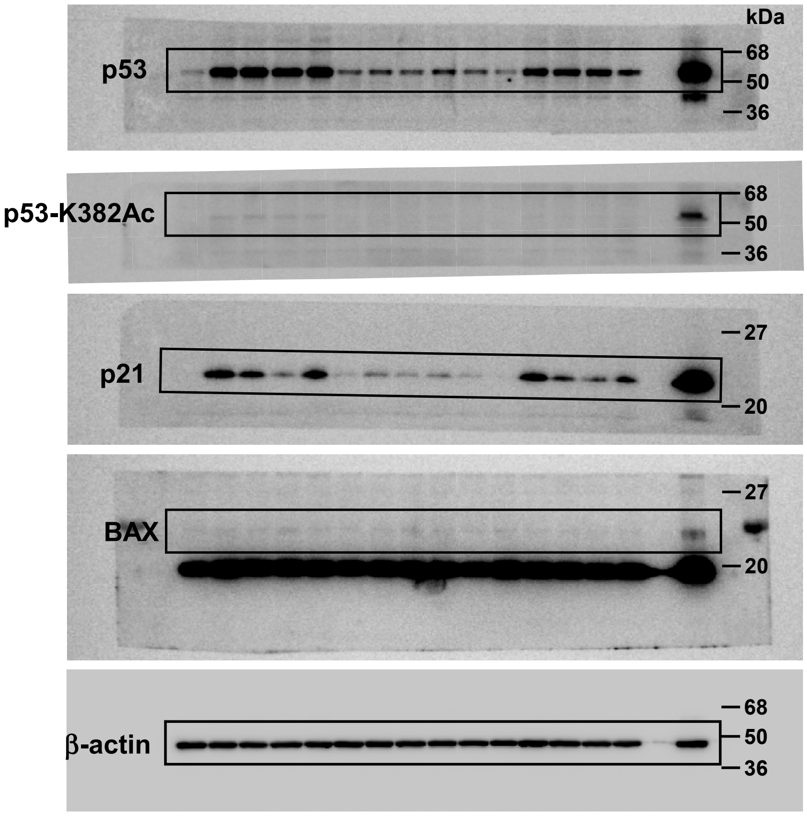

Supplementary Figure S22. complete scan of the blots presented in Figure 4g

Fig. 5b

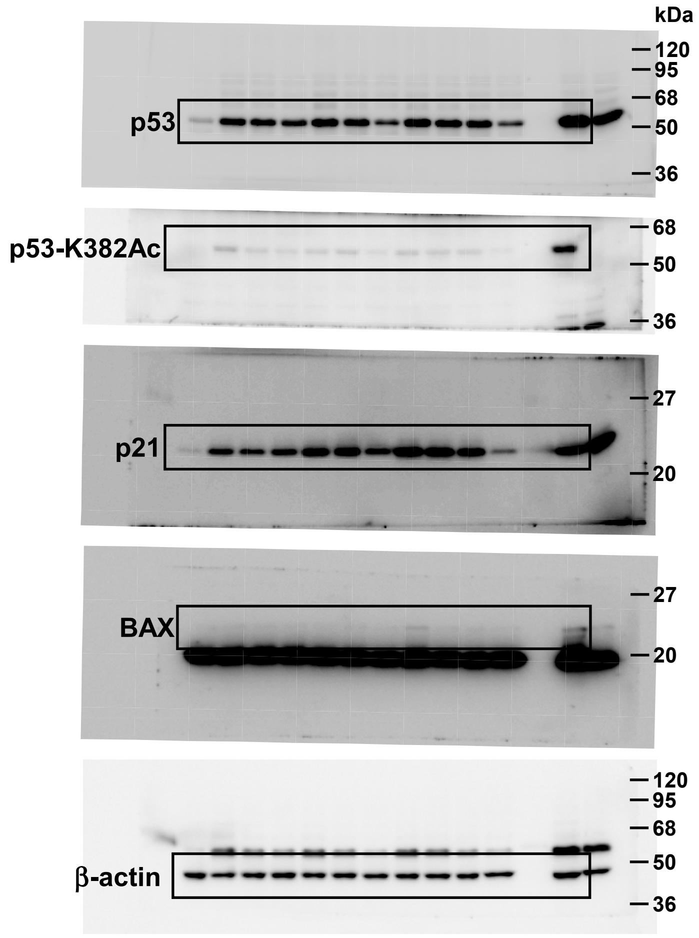

Supplementary Figure S23. complete scan of the blots presented in Figure 5b

Fig. 5d

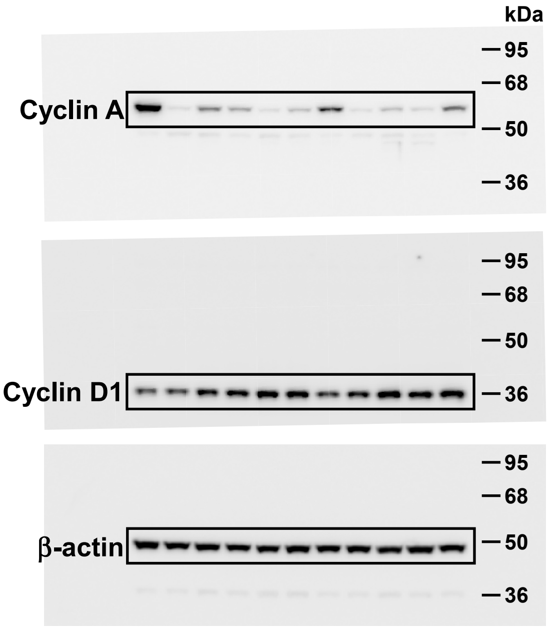

Supplementary Figure S24. complete scan of the blots presented in Figure 5d
